# Rhodopsin orphan GPCR20 interacts with neuropeptides and directs growth, sexual differentiation, and egg production in female *Schistosoma mansoni*

**DOI:** 10.1128/spectrum.02193-23

**Published:** 2023-12-04

**Authors:** Xuesong Li, Oliver Weth, Martin Haimann, Max F. Möscheid, Theresa S. Huber, Christoph G. Grevelding

**Affiliations:** 1 Institute for Parasitology, BFS, Justus Liebig University Giessen, Giessen, Germany; University of Wisconsin-Madison, Madison, Wisconsin, USA

**Keywords:** schistosomes, G protein-coupled receptor, neuropeptide, membrane-anchored ligand and receptor yeast two-hybrid system, neuronal signaling

## Abstract

Schistosomes are parasitic flatworms that cause schistosomiasis, a neglected tropical disease of worldwide importance. Since standard treatment of schistosomiasis relies on a single drug, praziquantel, alternative drugs are needed. G protein-coupled receptors (GPCRs) represent promising targets for new anthelmintics. Although GPCRs represent a prominent receptor class in schistosomes, functional studies are limited just as knowledge about their ligands. Candidate ligands are neuropeptides acting as neurotransmitters, neuromodulators, or hormones in the nervous system. Transcriptomics studies in *Schistosoma mansoni* indicated that nearly all neuropeptide genes (*Sm_npps*) and a subgroup of GPCRs exhibited a sex- and pairing-dependent expression profile. Among these was the rhodopsin orphan GPCR20 (*Sm*GPCR20), which we characterized in our study. Using a yeast two-hybrid-based approach, we identified specific interactions between *Sm*GPCR20 and two neuropeptides *Sm*NPP26 and *Sm*NPP40. As analyzed by qRT-PCR, *Smgpcr20*, *Smnpp26*, and *Smnpp40* showed sex- and/or pairing-influenced expression. Whole-mount *in situ* hybridization exhibited transcripts of these genes in neuronal cells, subtegumental area, and parenchyma of both sexes. Furthermore, we received indication for co-localization of transcripts of these genes in the anterior “head” region of single-sex females and in particular patterns along the worm body indicating neuronal expression. RNA interference (RNAi) with combinations of double-stranded RNAs against the three genes resulted in reduced egg production. Confocal microscopy revealed morphologic changes in the female gonads. Furthermore, RNAi in first-time paired females caused a reduced length of females after double knockdown of *Sm*GPCR20 and *Sm*NPP26 and changes in the ovary. In addition, we found reduced transcript levels of egg formation-associated and gonad-specifically transcribed genes and the stem-cell marker *nanos-1*. The obtained results suggest that *Sm*NPP26 and *Sm*NPP40 are potential ligands of *Sm*GPCR20 and that this GPCR in combination with both neuropeptides affects egg production, oogenesis, and growth of *S. mansoni* females.

**IMPORTANCE:**

Schistosomes cause schistosomiasis, one of the neglected tropical diseases as defined by the WHO. For decades, the treatment of schistosomiasis relies on a single drug, praziquantel. Due to its wide use, there is justified fear of resistance against this drug, and a vaccine is not available. Besides its biological relevance in signal transduction processes, the class of G protein-coupled receptors (GPCRs) is also well suited for drug design. Against this background, we characterized one GPCR of *Schistosoma mansoni*, *Sm*GPCR20, at the molecular and functional level. We identified two potential neuropeptides (NPPs) as ligands, *Sm*NPP26 and *Sm*NPP40, and unraveled their roles, in combination with *Sm*GPCR20, in neuronal processes controlling egg production, oogenesis, and growth of *S. mansoni* females. Since eggs are closely associated with the pathogenesis of schistosomiasis, our results contribute to the understanding of processes leading to egg production in schistosomes, which is under the control of pairing in this exceptional parasite.

## INTRODUCTION

Schistosomiasis is one of the most prevalent infectious diseases worldwide and caused by parasitic trematodes of the genus *Schistosoma*. Schistosomiasis has been recorded in 78 countries worldwide (1), and more than 250 million people are infected (1, 2). Currently, a vaccine is not available, and praziquantel (PZQ) represents today’s most effective drug against schistosomes (3). However, several reports discussed the possibility of resistance development in *Schistosoma mansoni* against PZQ (2, 4 – 7), which indicates the urgent need to find alternative treatment options.

The eggs of schistosomes are the pathogenic agents since they can cause inflammation and fibrosis in organs such as liver and spleen (2, 8, 9). Egg production, in turn, depends on an exceptional feature of schistosome biology, which is associated with pairing. Only upon a permanent pairing contact with a male, mitogenic activity and gonad differentiation are induced in the female. This leads to the complete development of the female gonads, ovary and the vitellarium, which provide all cells required for egg production (10, 11). Thus, schistosome female development is characterized by a high turnover of gonadal cell, which is tightly controlled by the male partner. Separation of couples leads to the loss of the reproductive capacity of the female, which coincides with dedifferentiation processes in its gonads. However, after re-pairing, the female gonads re-differentiate, and egg production is resumed (12). Although this phenomenon is long known (13), just recently a first factor was identified, β-alanyl-tryptamine, which is provided by the male to induce gonad differentiation processes in the female (14). Furthermore, biogenic amines have been suggested to participate in this process (15, 16). Beyond that, however, our knowledge of the male-induced sexual maturation of the schistosome female is still fragmentary.

G protein-coupled receptors (GPCRs) are the largest superfamily of integral transmembrane receptors. GPCRs are classified into five families, glutamate, rhodopsin, adhesion, frizzled/taste2, and secretin (17 – 19). Upon ligand binding and activation, GPCRs can control downstream processes such as growth, differentiation, and reproduction (20 – 25). As ligands, especially neuropeptides can activate GPCRs of the rhodopsin family. Previous studies in invertebrates provided evidence for the role of neuropeptide-GPCRs interaction in processes controlling germline stem-cell (GSC) proliferation to promote oocyte maturation (26 – 29). This included studies in *S. japonicum*, in which an allatostatin neuropeptide receptor was shown to regulate the development of reproductive organs in the paired female (14). Also, the glutamate receptor *Sj*GRM7 of *S. japonicum* was shown to be important for physiological activity, growth, development, and egg production (30).

Genome sequencing of *S. mansoni* revealed the existence of 126 GPCRs (25, 31). However, only few of these have been characterized at the molecular and functional levels (32 – 36). RNA-seq analyses comparing transcriptomes of paired vs unpaired females, males, and their gonads, respectively, indicated that many GPCRs were transcribed in non-gonad tissues in *S. mansoni* adults (37). Furthermore, for some of these GPCRs, the so-called bM-sM-sF subgroup, a pairing-influenced transcript occurrence was discovered with high transcript levels in paired males (bM), unpaired males (sM), and unpaired females (sF), whereas comparably low or no transcripts of these GPCRs occurred in paired females (bF) (25, 37). Remarkably, the majority of genes potentially coding for neuropeptide genes seemed to be regulated in a sex- and pairing-dependent manner, similar to the bM-sM-sF subgroup (38).

To shed first light on the importance of one GPCR member of the bM-sM-sF subgroup of *S. mansoni*, named *Sm*GPCR20, we performed a deorphanization approach to identify potential neuropeptide (*Sm-npps*) interaction partners. To this end, we made use of an established membrane-anchored ligand and receptor yeast two-hybrid system (MALAR-Y2H) (39, 40). Besides identifying *Sm*NPP26 and *Sm*NPP40 as potential ligands of *Sm*GPCR20, we functionally characterized these three genes. From our results, we conclude on a sex- and/or pairing-influenced expression of *Sm*GPCR20, *Sm*NPP26, and *Sm*NPP40. Functional analyses suggest roles of these three genes for growth, development, and egg production of paired *S. mansoni* females.

## RESULTS

### Sequence analyses of Smp_084270, Smp_071050, and Smp_004710

**Fig 1:**
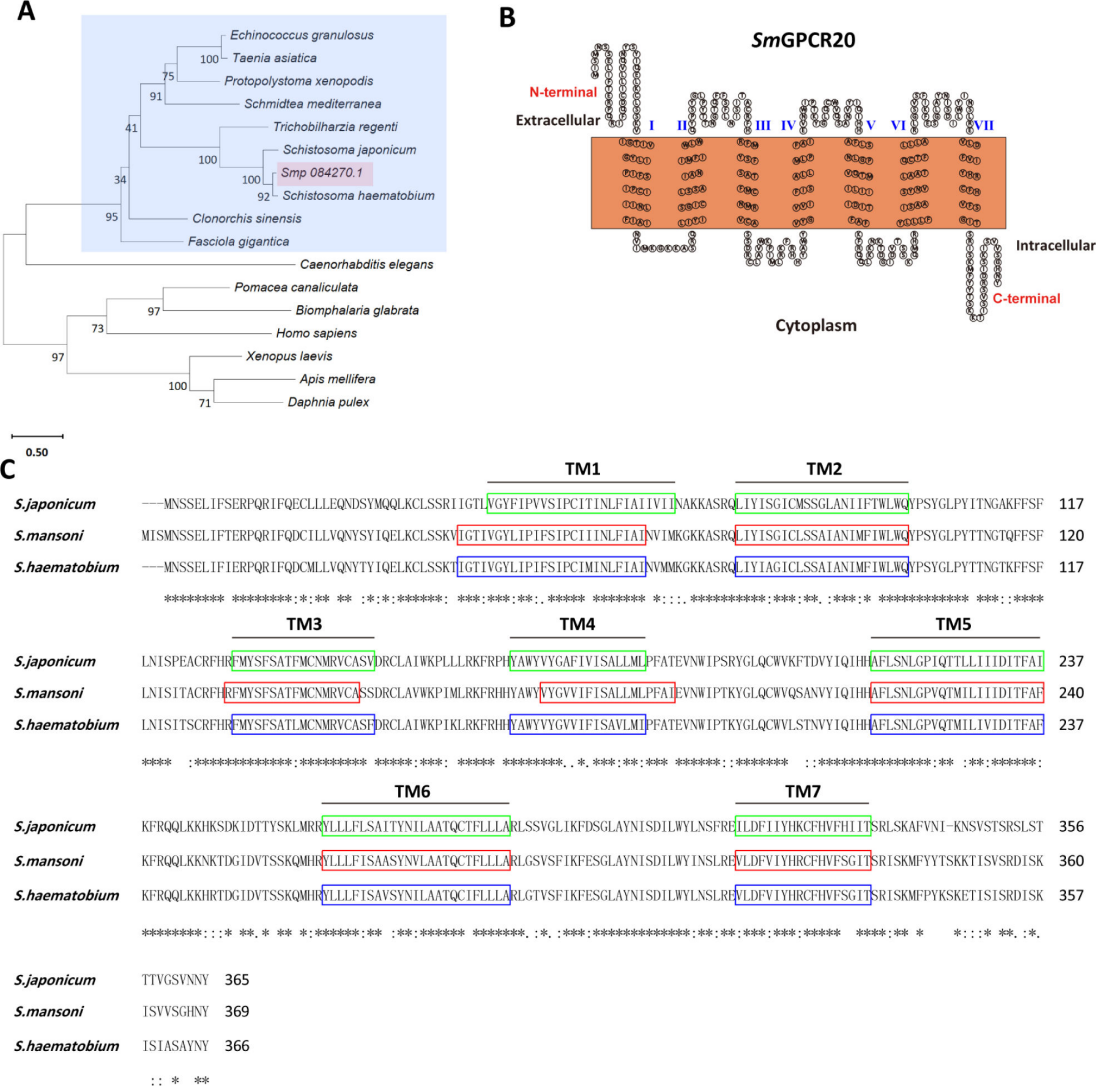
Bioinformatic analysis indicated that SmGPCR20 (Smp_084270) belongs to a family of platyhelminths-specific rhodopsin-like orphan GPCRs. (**A**) Phylogenetic analysis of Smp_084270. Sequences represented in the phylogenetic analysis originated from the trematodes *Schistosoma japonicum* (A0A4Z2CQX1), *S. mansoni* [Smp_084270 (A0A3Q0KIK8)], *S. haematobium* (A0A095C4R8), *Trichobilharzia regenti* (A0A183WMW7), *Clonorchis sinensis* (H2KRY6), and *Fasciola gigantica* (A0A504Z2P0), from the cestodes *Echinococcus granulosus* (U6J3B5) and *Taenia asiatica* (A0A0R3W0W9), from the monogenean parasite *Protopolystoma xenopodis* (A0A3S5CMM6), from the planaria *Schmidtea mediterranea* (A0A193KU74), from the nematode *C. elegans* (G5EDR2), from the molluscs *Pomacea canaliculata* (A0A2T7PVN7) and *Biomphalaria glabrata* (A0A2C9LEA4), from the arthropods *Apis mellifera* (Q9NG02) and *Daphnia pulex* (E9G3B6), from the amphibian *Xenopus laevis* (B7ZRQ1), and from *Homo sapiens* (Q14439). (**B**) The transmembrane domain composition of *Sm*GPCR20 as predicted by DeepTMHMM and SACS HMMTOP. (**C**) Multiple protein alignment of *Sm*GPCR20 and its orthologs of *S. haematobium* and *S. japonicum*. Highlighted are the 7 TM sequence motifs (TM1–TM7) (colored in green, red, and blue); * indicates sequence identity; : indicates sequence similarity.

The sequence of the GPCR gene Smp_084270 is available on the Uniprot website (https://www.uniprot.org/) under accession number A0A3Q0KIK8. *In silico* analysis indicated its sequence similarity to members of the GPCR_Rhodpsn_7TM protein family (access: IPR017452), and we named it *Sm*GPCR20. Phylogenetic analysis showed the existence of *Sm*GPCR20 orthologs in *S. japonicum*, *S. haematobium* (A0A095C4R8)*, Trichobilharzia regenti*, *Clonorchis sinensis*, *Fasciola gigantica*, *Schmidtea mediterranea*, *Echinococcus granulosus, Taenia asiatica,* and *Protopolystoma xenopodis*. They form a GPCR20 helminth clade, which diverges from the nematode *Caenorhabditis elegans* and further invertebrates and vertebrates (Fig. 1A; Fig. S1). DeepTMHMM and SACS HMMTOP analyses predicted 7 TM domains in *Sm*GPCR20 (Fig. 1B). Finally, multiple sequence alignments showed high sequence similarities to the orthologs of *S. japonicum* (A0A4Z2CQX1) (76.44% identity) and *S. haematobium* (A0A095C4R8) (90.71% identity). The predicted 7 TM domains were highly conserved between *S. mansoni* and *S. haematobium* (Fig. 1C).

*Sm*NPP26 (Smp_071050) and *Sm*NPP40 (Smp_004710) are peptide prohormone genes. According to previous analyses, both NPPs represent novel members of neuropeptide families predicted to occur in parasitic flatworms with orthologs in their free-living relative *Schmidtea mediterranea* (26, 41). Both genes may code for multiple distinct peptides (Fig. S2).

### MALAR-Y2H analysis identified neuropeptides *Sm*NPP26 and *Sm*NPP40 as potential interaction partners of *Sm*GPCR20

**Fig 2:**
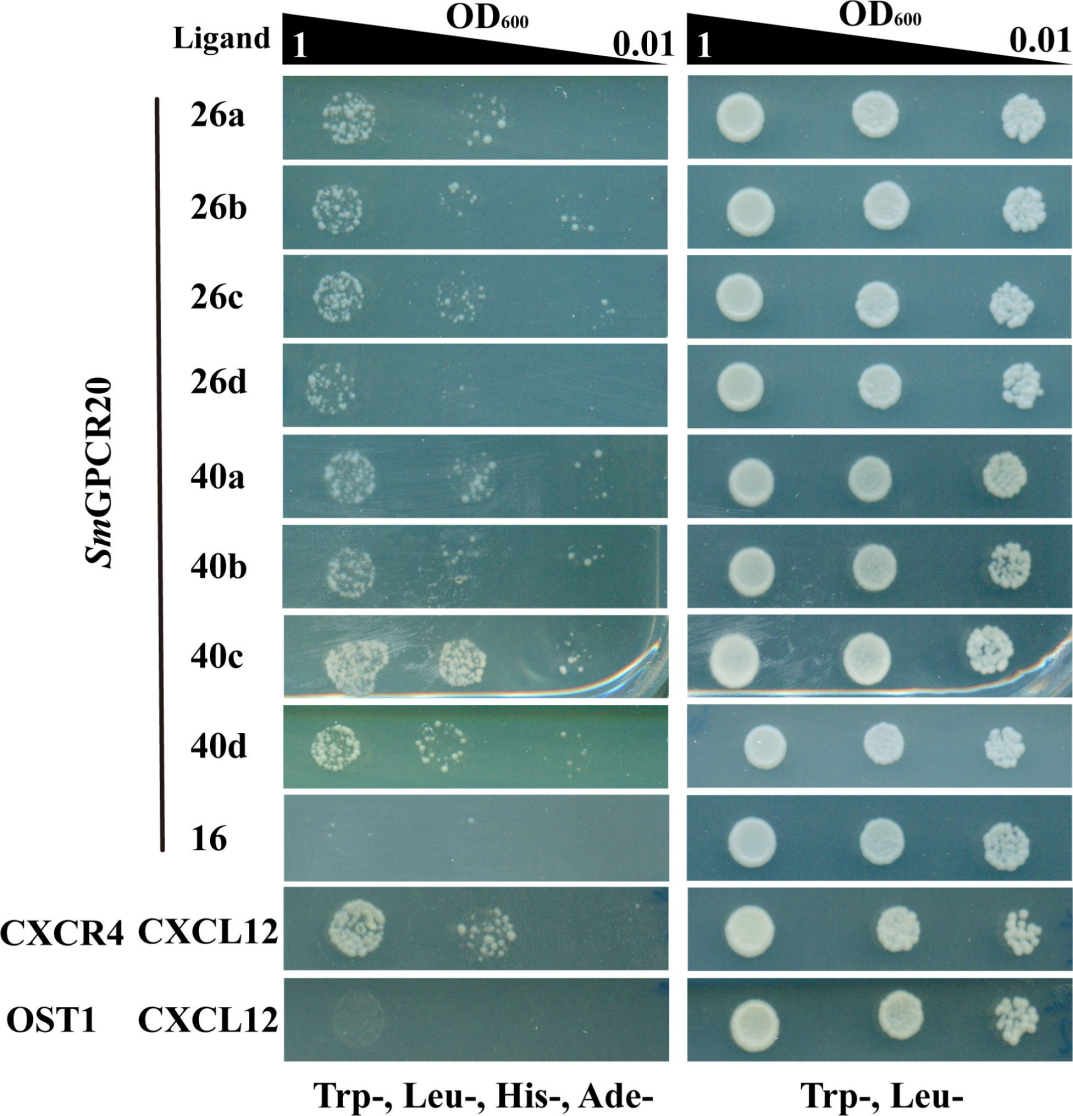
The MALAR-Y2H identified neuropeptide interaction partners of *Sm*GPCR20. Shown are cell-growth assays of the yeast strain AH109 transfected with plasmids expressing the ligand fusion proteins (40). These cells were mated with yeast strain Y187, which had been transfected with a plasmid expressing *Sm*GPCR20 as fusion protein. The Trp^−^, Leu^−^ control (right panel) indicated the successful transformation of yeast cells with all used plasmids. Trp^−^, Leu^−^, His^−^, and Ade^−^ (left panel) showed growth under selection conditions for protein interaction, in this case with the candidate neuropeptides L26 and L40 (a, b, c, and d indicate different fragments of *Sm*NPP26 and *Sm*NPP40). The chemokine CXCL12 and its known receptor CXCR4 were employed as positive control. OST1 is a transmembrane protein of *S. cerevisiae* and, in combination with CXCL12, used as the negative control (39).

To identify ligands of *Sm*GPCR20, we employed the MALAR-Y2H system that was previously shown to be suitable for ligand identification of schistosome GPCRs (40). To this end, *Sm*GPCR20 was cloned as fusion protein with the N-terminal part of ubiquitin, and the resulting construct transformed into yeast strain Y187. As potential ligands, 47 neuropeptides each were fused via a flexible linker to the WBP1 transmembrane domain of yeast for membrane integration (with the neuropeptide part as extracellular domain) and to the C-terminal part of the split ubiquitin system (as intracellular part). We transformed these constructs (individually) into yeast strain AH109 and used SD-Leu/-Trp to select mated clones co-expressing bait and prey constructs simultaneously. Using SD-Leu/-Trp/-His/-Ade plates, we further selected for interaction of the *Sm*GPCR20 receptor and presumptive ligands. Of the 47 neuropeptide candidates, only 2 induced growths under selection conditions in diploid yeasts co-expressing *Sm*GPCR20. These two neuropeptides were *Sm*NPP26 (Smp_071050) and *Sm*NPP40 (Smp_004710) (Fig. 2). Only weak or no growth was monitored for all other 45 combinations (exemplified by *Sm*NPP16 as representative NPP probe) (Fig. 2). As expected, the positive control (CXCR4/CXCL12) (39) showed growth comparable to *Sm*GPCR20/*Sm*NPP26 (a, b, c, and d) or *Sm*GPCR20/*Sm*NPP40 (a, b, c, and d) (Fig. 2; a–d indicate different fragments of *Sm*NPP26 and *Sm*NPP40). As expected, we observed no growth on the selective medium with the negative control (OST1/CXCL12) (39). These results suggested specific interactions between *Sm*GPCR20/*Sm*NPP26 and *Sm*GPCR20/*Sm*NPP40.

### *Sm*GPCR20, *Sm*NPP26, and *Sm*NPP40 are expressed in *S. mansoni* adults, mainly in neuronal tissues

**Fig 3:**
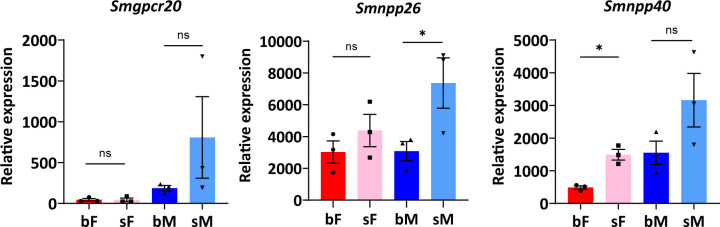
Transcriptional profiles of *Sm*GPCR20, *Sm*NPP26, and *Sm*NPP40 in bM, bF, and sF as determined by real-time PCR. Relative expression levels of transcripts were analyzed by the 2^−△Ct^ method, using the formula: relative expression = 2^−∆Ct^ × *f*, with *f =* 1,000 as an arbitrary factor (42). Data are representative of the mean  ±  SEM of three separate experiments. Significant differences were determined by two-way ANOVA or one-way ANOVA, indicated as *P < 0.05.

To characterize these three genes at the molecular level, we first investigated their transcriptional profiles by qRT-PCR. The results showed expression of all genes in bF [paired (bisex, bF) females], sF [unpaired (single-sex, sF) females], bM [paired (bisex, bM) males], and sM [unpaired (single-sex, sM) males] (Fig. 3). For *Smgpcr20*, slightly higher transcript levels were found in sM compared with bM and similar low levels in sF and bF (Fig. 3). The results corresponded to previous RNA-seq results of gene expression profiles of paired and unpaired female and male *S. mansoni* (Fig. S3) (37). For *Smnpp26* and *Smnpp40*, we obtained similar sM > bM transcript patterns. A similar tendency (sF > bF) was also detected for females (Fig. 3). The female data matched to previous RNA-seq results, whereas these former data showed no obvious pairing influence for both *npps* in males (Fig. S3) (37).

**Fig 4:**
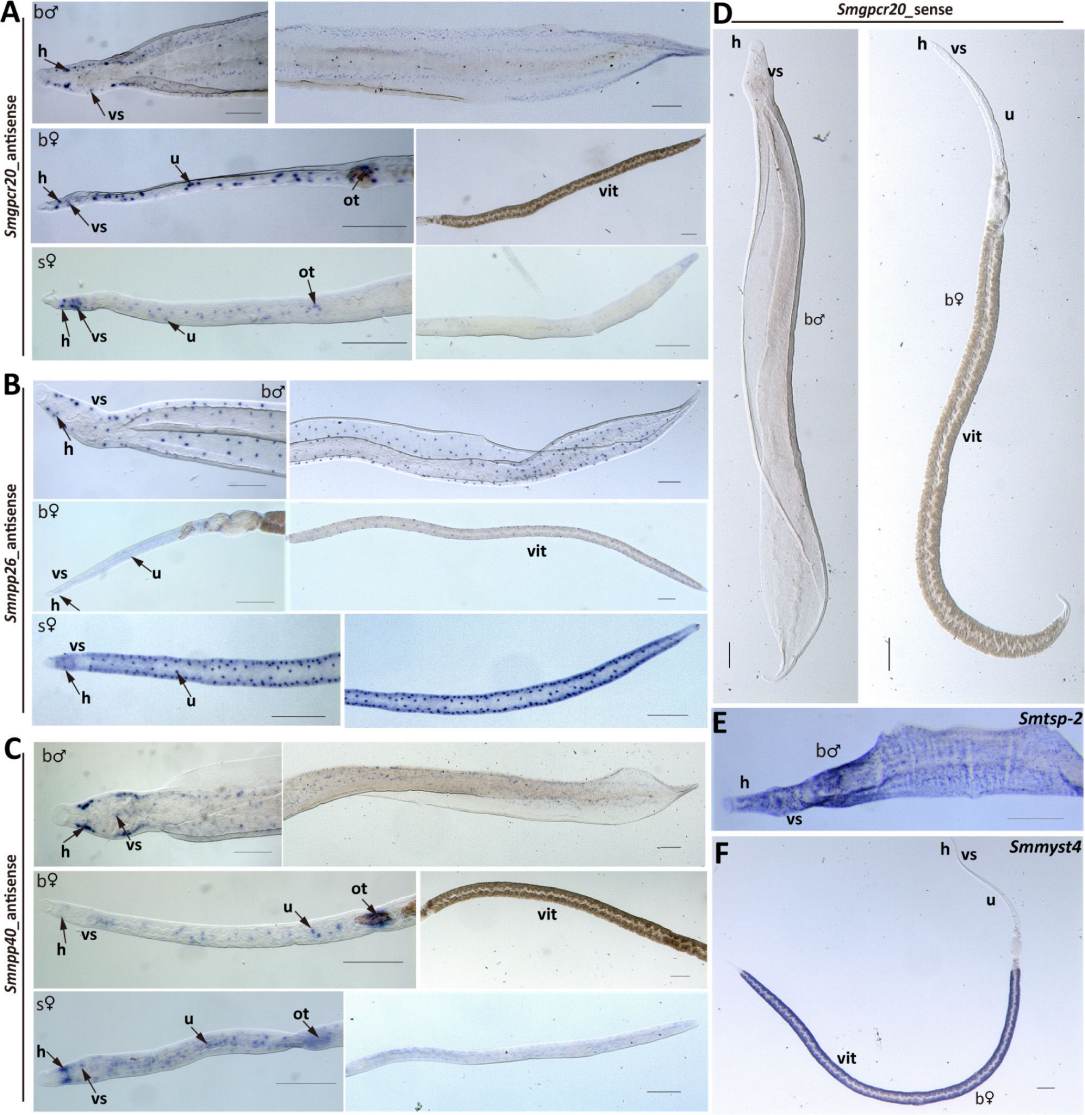
WISH analyses revealed localization of *Smgpcr20, Smnpp26,* and *Smnpp40* mainly in neuronal cells. A collection of WISH results showing the expression of *Smgpcr20* (**A**), *Smnpp26* (**B**), and *Smnpp40* (**C**) in bM, bF, and sF. Sense probes served as negative controls and showed no signals (**D**). As positive control, we used a probe detecting *Smtsp-2* (**E**), a tetraspanin gene with known expression pattern along tegument of *S. mansoni* (44). *Smmyst4* (37; Möscheid et al., unpublished data), a vitellarium marker, was used as a positive control for the vitellarium-specific transcripts in bF (**F**). In some figure parts such as panel B, sF, adjacent images show different parts of the same flatworm to better illustrate the pervasive presence of stained cells (or signal-free areas such as in panel C, bF, vit) throughout the organism. vit, vitellarium; ov, ovary; u, uterus; ot, ootype; h, head; vs, ventral sucker. Scale bars = 200 µm.

To localize the transcripts of these genes, we performed whole-mount colorimetric *in situ* hybridization (WISH) with bM, bF, and sF. Especially for the neuropeptides, we noted increased numbers of signals in bM and sF compared with bF and for *Smnpp26* in bM and sF throughout their worm bodies (Fig. 4A through C). Moreover, we observed distinct and similar, dot pattern-like signals for all three transcripts. Their occurrence resembled neuronal expression patterns as shown previously and supported by RNA-seq data (Fig. S4) (43). In bM and sF, these signals occurred along the body (Fig. 4A through C). Remarkable differences were observed comparing sF and bF. In sF, signals were nearly evenly distributed along the whole body, whereas in bF, the signals dominated in the anterior part along the uterus and the ootype, which was especially evident for *Smgpcr20* and *Smnpp40* (Fig. 4A and C). In the vitellarium of bF, we observed no signals of these two genes. However, *Smnpp26* transcript signals were distributed along the whole body of bF, which was similar to sF (Fig. 4B). In the posterior part of bF, it seemed that these signals occurred in the subtegumental region along the vitellarium but not within the vitellarium. As negative controls, we used sense probes of both genes, and no signals were detected (Fig. 4D; Fig. S5). As positive control for males, we used a probe detecting transcripts of the tetraspanin gene (*Smtsp-2*) of *S. mansoni* (Fig. 4E), which is transcribed along the tegument area (44, 45). Smp_165360, a presumptive vitellarium marker (*Smmyst4*) (37; Möscheid et al., unpublished data), was used as a positive control for bF. As expected, Smp_165360 transcripts were detected in the vitellarium (Fig. 4F).

**Fig 5:**
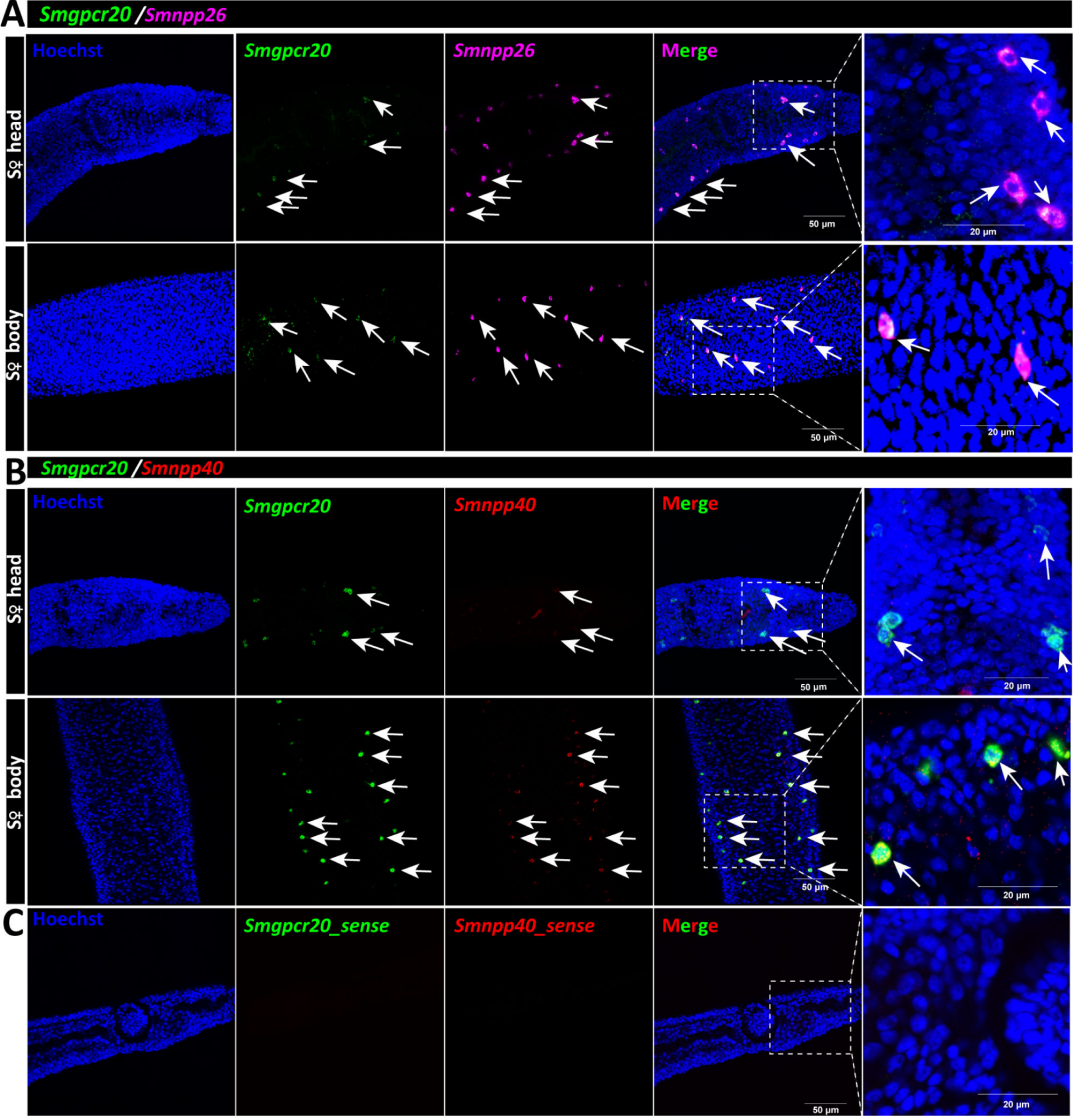
Double FISH analyses revealed colocalization of *Smgpcr20, Smnpp26, and Smnpp40*. Results of double FISH experiments providing first hints for *Smgpcr20* and *Smnpp26* transcript colocalization in neuronal cells within the head region and along the body of sF. For counterstaining, cells were labeled by Hoechst 33342 (blue). FITC-labeled *Smgpcr20* transcripts are shown in green, and DIG-labeled *Smnpp26* transcripts are shown in magenta. (A) Results of double FISH experiments showing *Smgpcr20* and *Smnpp26* transcript colocalization in the head and body regions of sF. (**B**) Results of double FISH experiments showing *Smgpcr20* and *Smnpp40* transcript colocalization in the head and body regions of sF. Sense probes of each gene served as negative controls and showed no signals (**C**). Cells were stained Hoechst 33342 (blue), FITC-labeled *Smgpcr20* transcripts are shown in green, and DIG-labeled *Smnpp40* transcripts are shown in red. Scale bars: 50 µm; right panel: 20 µm.

Next, we investigated the potential co-localization of *Smgpcr20* with *Smnpp26* and *Smnpp40*, by double fluorescence *in situ* hybridization (FISH) experiments in sF. To this end, an antisense RNA probe labeled by fluorescein isothiocyanate (FITC) was used for *Smgpcr20* FISH, whereas *Smnpp26* and *Smnpp40* antisense RNA probes were labeled by DIG. In the head region and along the body of sF, we found hints for co-localization of transcripts of *Smgpcr20/Smnpp26* (Fig. 5A) and *Smgpcr20/Smnpp40* (Fig. 5B). As negative controls, we used sense probes of each gene and detected no signals (Fig. 5C).

### RNAi against *Sm*GPCR20, *Sm*NPP26, and *Sm*NPP40 indicated functions for egg production and ovary structure of *S. mansoni*

To explore the potential functions of *Sm*GPCR20, *Sm*NPP26, and *Sm*NPP40, we performed RNAi experiments. To this end, *S. mansoni* couples were treated with double-stranded RNAs (dsRNAs) against all three genes in individual knockdown (KD) experiments *in vitro*. Primary RNAi experiments against either *Sm*GPCR20, or *Sm*NPP26, or *Sm*NPP40 showed trends toward reduced egg production of bF but in no case significant effects on this or other physiological parameters such as motility, viability, and pairing stability (not shown). Since KD efficiencies varied in these pilot experiments, we assumed that simultaneous RNAi against *gpcr20* and both *npps* may amplify potential KD effects. Therefore, we tested combinations of dsRNAs against *Smgpcr20/Smnpp26*, and *Smgpcr20*/*Smnpp40*, or a combination of all three gene-specific dsRNAs. During the experimental period of 15 days, we determined the abovementioned physiological parameters every 2–3 days by bright-field microscopy. Compared with two control groups (kept under the same *in vitro* conditions but either without dsRNA or treated with a non-schistosome dsRNA as negative control), the transcript levels of all three genes decreased significantly, by >70% for *Smgpcr20*, by >90% for *Smnpp26*, and by >80% for *Smnpp40* (Fig. S6).

**Fig 6:**
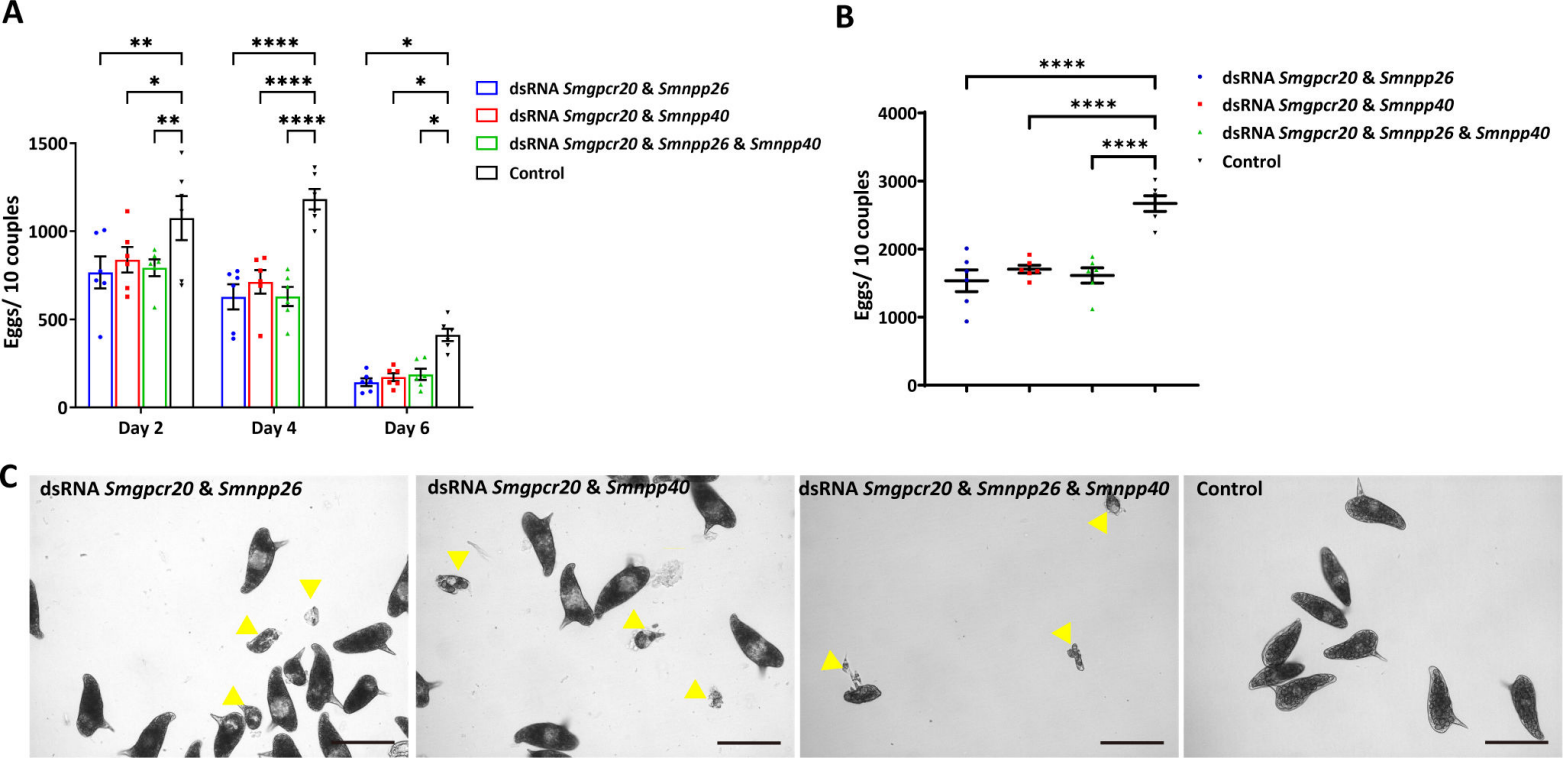
RNAi against *Smgpcr20*, *Smnpp26*, and *Smnpp40* negatively affected egg production in *S. mansoni*. The number of eggs laid in the first 6 days (**A**) and the total number of eggs (**B**) laid during the observation period of 15 days declined in *S. mansoni* couples treated with dsRNA combinations of *Smgpcr20* and *Smnpp26*, *Smgpcr20* and *Smnpp40* dsRNA, or a combination of three gene-specific dsRNAs. The points are representative for the single experiments. (C) After dsRNA treatments with *Smgpcr20*/*Smnpp26*, *Smgpcr20*/*Smnpp40*, or a combination of the three gene-specific dsRNAs, besides normal eggs, deformed eggs were also found from day 6 on, but their numbers were not significantly different in the RNAi groups compared to the control group (F; see also Fig. S8D). Data in A and B mean  ±  SEM of three separate experiments (three biological, each with two technical replicates). Significant differences in panels A and B were determined by two-way ANOVA or one-way ANOVA, indicated as ****P < 0.0001, **P < 0.01, *P < 0.05. Scale bars (**C–F**): 100 µm.

Although the overall number of couples of the treatment groups was slightly lower than the control, pairing stability of the couples was not affected among the treatment groups during the observation period (Fig. S7). Furthermore, we observed no effects of the different dsRNA treatments on worm motility and vitality. However, compared with the control groups, females treated with dsRNA combinations of *Smgpcr20* and *Smnpp26*, *Smgpcr20* and *Smnpp40*, or a combination of all three dsRNAs exhibited a decline in egg production from day 2 on. After 6 days, the levels of egg production decreased about 50%–60% in all treatment groups (Fig. 6A). Correspondingly, the total number of eggs laid during the whole treatment period declined about 40% compared to the control (Fig. 6B). The occurrence of deformed eggs was observed from day 6 on (Fig. 6C through E); however, their number increased over time and was not significantly different in the triple KD group compared to the control groups (Fig. S8).

**Fig 7:**
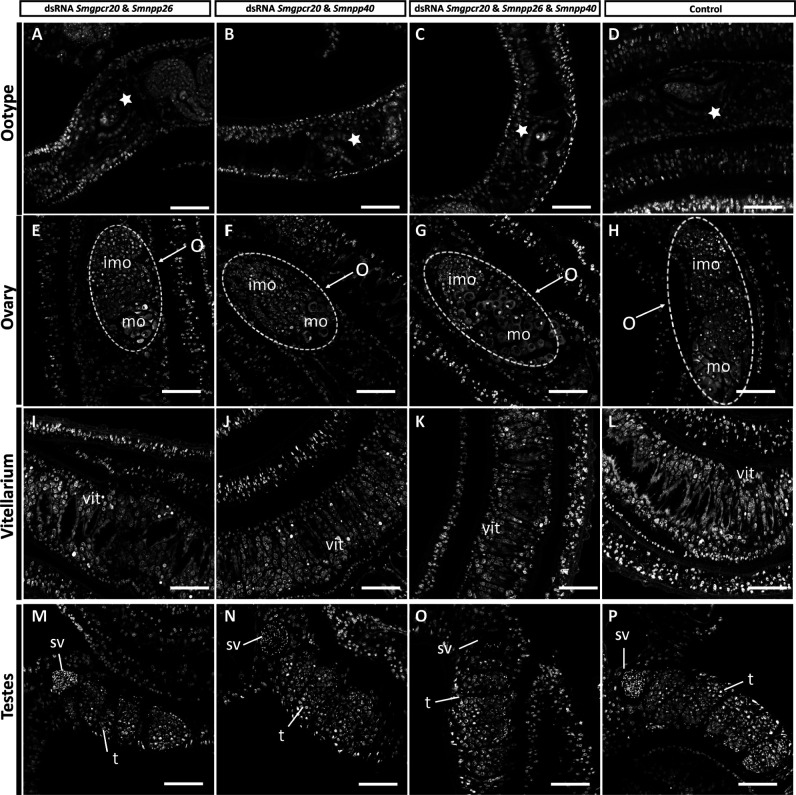
RNAi induced morphologic changes in ovaries of female *S. mansoni*. CLSM analysis of *S. mansoni* females and males treated for 15 days with different combinations of dsRNA (as indicated): (A, E, I, and M) *Smgpcr20* and *Smnpp26* dsRNA; (B, F, J, and N) *Smgpcr20* and *Smnpp40* dsRNA; (C, G, K, and O) a combination of the three gene-specific dsRNAs; (D, H, L, and P) control worms maintained under the same culture conditions but without dsRNA (*n* = 5 per experiment). O, ovary; imo, immature oocytes; mo, mature oocytes; sv, sperm vesicle; t, testes; vit, vitellarium; white stars indicate the ootype of females. Scale bars: 50 µm.

Furthermore, we performed morphological analyses by confocal laser scanning microscopy (CLSM) (Fig. 7). Whereas the ootype appeared unaffected (Fig. 7A through D), the length and width of the ovary of females of the treatment groups were smaller than those of the untreated control, which might have resulted from a decreased number of mature oocytes (in the double-treatment groups) and a decreased number of immature oocytes in the triple-treatment group (Fig. 7E through G). In a control group (without dsRNA), the ovary exhibited large mature oocytes in the posterior part and small, stem cell-like oogonia (immature oocytes) in the anterior part (Fig. 7H). However, no obvious morphological changes were detected in the vitellarium of females in the treatment groups (Fig. 7I through K), compared to the control (Fig. 7L).

Since a high amount of dsRNA (30 µg/mL for *Smgpcr20* and 15 µg/mL for *Smnpp26* and *Smnpp40*, respectively) was used for the triple KD approach, we performed another control experiment to exclude that treatment with this high amount of dsRNA may negatively influence our results. To this end, we made use of dsRNA of the *ampR* gene of *Escherichia coli*, which in a recent study was shown to be suitable as “irrelevant” control dsRNA in RNAi experiments with *S. mansoni* (46). Since in the mentioned study, 30 µg/mL dsRNA had been used, we repeated the experiment here with 60 µg/mL dsRNA. Compared with the dsRNA^−^ control, treatment of couples with *ampR* dsRNA caused no significant effects on (i) the KD efficiencies of the genes in focus (Fig. S8A), (ii) on pairing stability (Fig. S8B), (iii) the amount of produced eggs and deformed eggs (Fig. S8C and D), (iv) stem-cell proliferation in the female ovary as determined by EdU labeling (Fig. S8E), and (v) ovary size as determined by CLSM Z-stacks and Image J analysis (Fig. S8F). Finally, the length of first-time paired females treated with 60 µg/mL *ampR* dsRNA showed no significant difference to the dsRNA^−^ control group (Fig. S8G). In contrast, in all cases, significant differences were observed between each of the two control groups and the triple KD group.

Finally, we looked for morphological changes also in males and their gonads by CLSM. No obvious morphologic changes were detected, neither along the male worm body nor in the testes structure (number and size of the testicular lobes), nor in the seminal vesicle, which was filled with differentiated sperm in all groups (Fig. 7M through O). Testes and seminal vesicles of males of the treatment groups showed no differences to control males (without dsRNA), which contained seminal vesicles filled with sperm and testicular lobes filled with numerous spermatogonia and spermatocytes at different stages of maturation (Fig. 7P).

### *Sm*GPCR20*, Sm*NPP26, and *Sm*NPP40 are required for different male-induced developmental processes in first-time paired females

**Fig 8:**
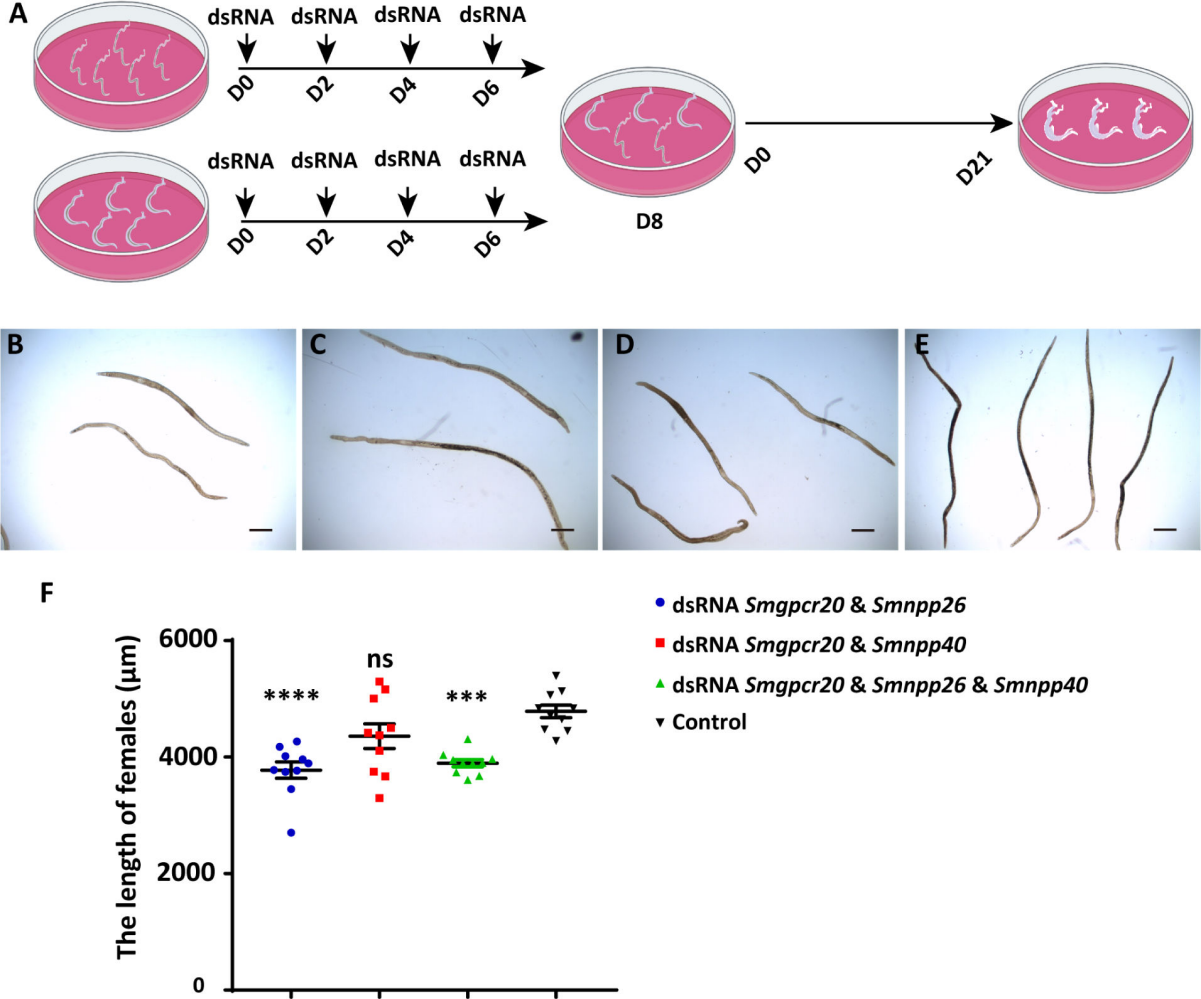
RNAi against *Smgpcr20*, *Smnpp26*, and *Smnpp40* affected growth of first-time paired *S. mansoni* females *in vitro*. (**A**) Schematic overview of the experiment to evaluate RNAi-mediated KD effects of *Smgpcr20*, *Smnpp26*, and *Smnpp40* on first-time paired females. To this end, single-sex females (without previous pairing experience) and mature males (with previous pairing experience) were separately maintained in Petri dishes, and dsRNA was added at days: D0, D2, D4, and D6. At day 8, the worms were combined and allowed to pair. Couples formed within 48 h and were maintained *in vitro* until day 21 (*n* = 3). (**B–E**) Representative examples of first-time paired females monitored by bright-field microscopy at day 21 of the experiment following treatment with *Smgpcr20*/*Smnpp26* dsRNAs (**B**), *Smgpcr20*/*Smnpp40* dsRNAs (**C**), *Smgpcr20*/*Smnpp26/Smnpp40* dsRNAs (**D**), and the untreated control (**E**). Scale bars: 500 µm. (**F**) Results of the determination of worm lengths (ImageJ-based) of the different RNAi groups (as indicated) and the untreated control (no dsRNA addition). The points are representatives for each of the 10 measurements of 10 individual females. ****P < 0.0001, ***P < 0.001.

Next, we investigated whether *Sm*GPCR20, *Sm*NPP26, and *Sm*NPP40 may also influence male-stimulated growth and development of first-time paired females. To this end, we performed RNAi in sF and bM for 8 days and then allowed pairing of these dsRNA-treated worms; sF and bM were treated with 15–30 μg/mL dsRNA of each target gene. Following pairing, which occurred within 2 days under conditions reported before (16), we maintained the couples for 21 days in culture without further dsRNA treatment (Fig. 8A). Compared with the control groups, the transcript levels of all three genes decreased significantly (Fig. S8G and S9), which was comparable to the KD approach with pairing-experienced females (Fig. S6). At day 21, for each group, 10 females were manually separated from their male partners and used for length determination by ImageJ (Fig. 8B through E). Compared to the control groups, the length of first-time paired females was significantly reduced upon treatment with the dsRNA combination *Smgpcr20*/*Smnpp26* (Fig. 8B) and the combination of all three dsRNAs (Fig. 8D and F; Fig. S8G). Here, the lengths of females were shortened by 21.1% (double KD) and 18.6% (triple KD), respectively (Fig. 8F). In contrast, the length of first-time paired females was only slightly reduced upon treatment with the dsRNA combination *Smgpcr20*/*Smnpp40* (Fig. 8C and F).

**Fig 9:**
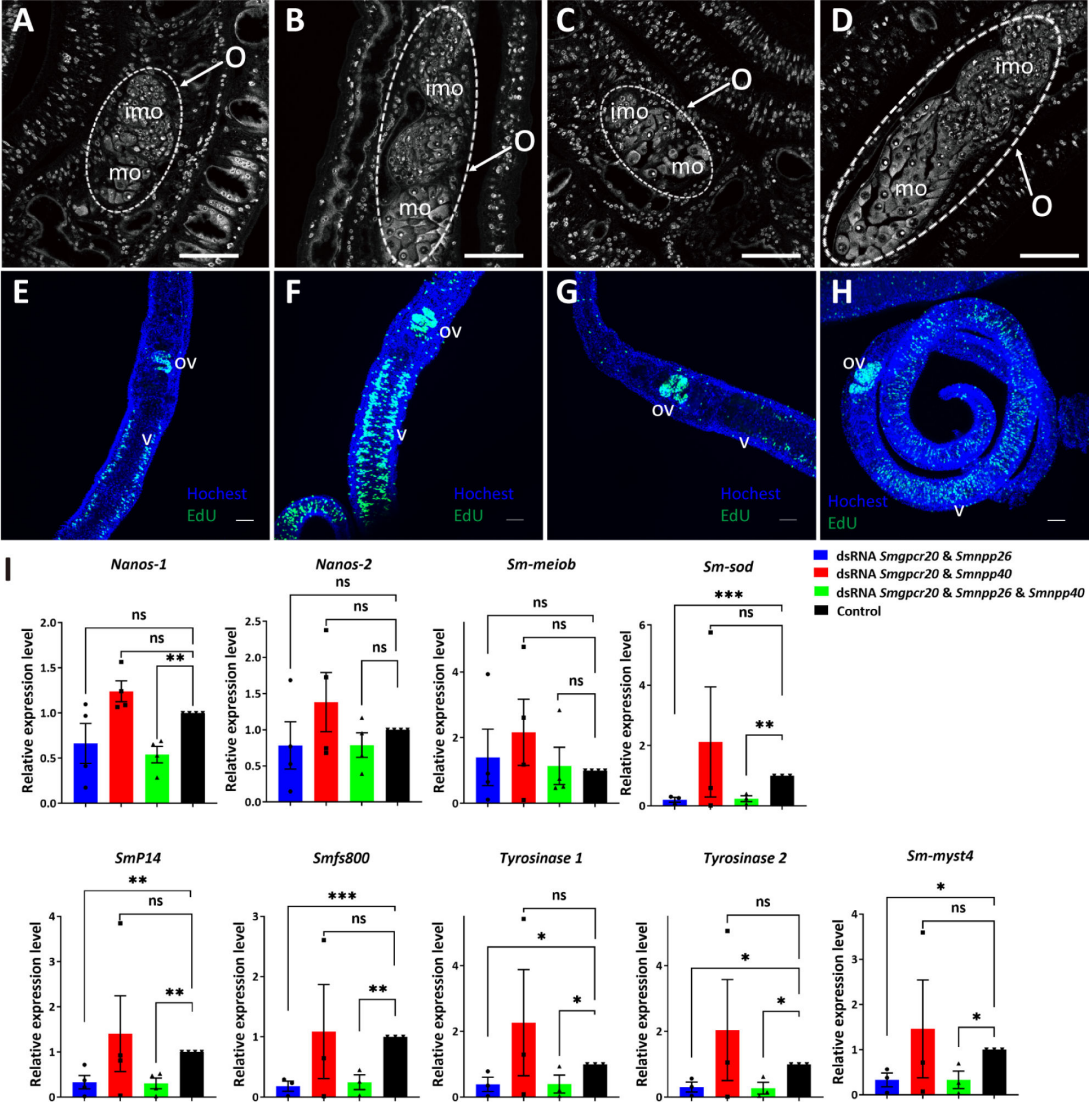
RNAi against *Smgpcr20*, *Smnpp26*, and *Smnpp40* affected gonad development, oocyte differentiation, and gene expression in first-time paired *S. mansoni* females. CLSM analysis showing representative pictures of *S. mansoni* females post pairing for 21 days (see Fig. 9A) in the presence of *Smgpcr20*/*Smnpp26* dsRNA (**A**), *Smgpcr20*/*Smnpp40* dsRNA (**B**), or a combination of *Smgpcr20*/*Smnpp26*/*Smnpp40* dsRNAs (**C**). First-time paired females maintained under the same conditions but without dsRNA served as control (**D**) (*n* = 5). O, ovary; imo, immature oocytes; mo, mature oocytes; scale bars: 50 µm. (**E–H**) Exemplary results of EdU staining of paired females (five each per experiment) following dsRNA treatment; Hoechst 33342 was used as counterstaining (blue), ov, ovary; v, vitellarium. The order of E–H is as in A–D. Compared to the control (**H**), a reduction of the number of EdU-stained cells was mainly observed in the *Smgpcr20*/*Smnpp26* (**A**) and the triple dsRNA treatment groups (**C**). (**I**) qRT-PCR experiments were performed to determine the transcript levels of selected genes. Fold changes of gene expression levels were calculated using the 2^−∆∆Ct^ method. The transcript levels of genes encoding *nanos-1*, *nanos-2, Sm*p14, fs800, *Smtyr*1, *Smtyr*2, *Smmyst*4, and *Smsod* were significantly lower than in the control group; *nanos-2* and *Smmeiob* transcript levels showed no significant differences among the RNAi and the control groups (*n* >3). Data are representatives of the mean  ±  SEM of three separate experiments. Significant differences determined by *t*-test were indicated as: ****P < 0.0001, **P < 0.01, *P < 0.05.

Furthermore, CLSM analyses showed that the dsRNA combination *Smgpcr20*/*Smnpp26* and the combination of all three dsRNAs resulted in poorly developed ovaries (Fig. 9). Compared to ovaries of first-time paired females kept under the same conditions *in vitro* but without adding any dsRNA (Fig. 9D), ovaries of treated females were reduced in size and exhibited fewer numbers of large mature oocytes in the posterior part and fewer immature oocytes in the anterior part of the ovary (Fig. 9A and C). Only a slight reduction of the number of mature oocytes was observed in paired females treated with the dsRNA combination *Smgpcr20*/*Smnpp40* but no effect on immature oocytes (Fig. 9B).

Next, we investigated potential effects of the dsRNA treatments on stem-cell proliferation by EdU incorporation experiments (Fig. 9E through H). Compared to the controls, we detected a clear reduction of the number of EdU-stained cells upon *Smgpcr20*/*Smnpp26* double KD and the triple KD (Fig. 9E and G; Fig. S8E). This points to reduced numbers of proliferating cells, which mainly affected the vitellarium, and in part also in the ovary. In contrast, no changes were observed in gonad of male worms (Fig. S10).

Since the dsRNA-induced phenotypes in pairing-experienced and first-time paired females indicated negative effects on egg production and poorly developed ovaries of paired females, we finally analyzed potential effects of RNAi on the transcript level of selected genes known to be involved in the egg production of *S. mansoni*. Among these genes were the egg-shell precursor genes *Smp14* (Smp_131110) (47), *fs800*-like (Smp_000270) (48), and the egg shell-forming genes tyrosinase 1 (*Sm*TYR1; Smp_050270) (49), tyrosinase 2 (*Sm*TYR2; Smp_013540) (49, 50), the GSC marker *nanos-1* (Smp_055740) (51, 52), the neoblast marker *nanos-2* (Smp_051920) (53), the GSC progeny marker *Smmeiob* (meiosis-related protein, Smp_333540; Möscheid et al., unpublished data), the extracellular superoxide dismutase (Cu-Zn) (*Sm*SOD; Smp_095980) (45, 54), and the histone acetyltransferase ortholog *myst4* (*Smmyst4*; Smp_165360) (37; Möscheid et al., unpublished data). To this end, we extracted RNA from first-time paired, dsRNA-treated females and control females (untreated), after manual separation from males. Afterward, we performed qRT-PCR analyses using the reference gene *Smletm1*, which was shown before to be a suitable control for this kind of *in vitro* culture experiments (55). The transcript levels of *Smp14, fs800, Smtyr1,* and *Smtyr2* were significantly decreased upon treatment with the dsRNA combination *Smgpcr20*/*Smnpp26* and the combination of all three dsRNAs (Fig. 9I). Only upon combining all three dsRNAs, the transcript level of *nanos-1* was significantly decreased (Fig. 9I). In contrast, the transcript levels of *nanos-2* and the GSC progeny marker *Smmeiob* were not downregulated following RNAi (Fig. 9I). We also found a significant decrease in the transcript levels of *Smmyst4* (a putative vitellarium marker) (37; Möscheid et al., unpublished data) and *Smsod* in dsRNA combinations *Smgpcr20*/*Smnpp26* and all three dsRNAs. In contrast, we found no significant difference in the transcript levels of all selected genes between the control (without dsRNA) and dsRNA combination *Smgpcr20*/*Smnpp40*.

## DISCUSSION

GPCRs are the largest superfamily of transmembrane receptors, and they have been proven as suitable drug targets in different organisms, including helminths (56 – 62). Some studies in *S. mansoni* have reported about GPCR involvement in neuromuscular processes (63), reproduction (25), and chemosensation (64). Former transcriptomic studies in references (37, 43, 65) indicated the existence of 116–117 GPCRs in *S. mansoni*, including a platyhelminth-specific rhodopsin subfamily, while a recent update pointed to 126 GPCRs (31). Except *S. haematobium*, which appeared to host a similar GPCR repertoire as *S. mansoni* (66), not much is known about GPCRs in other schistosome species. Although some GPCRs of *S. mansoni* have been characterized as receptors responding to histamine (67), dopamine (32, 68), glutamate (33, 69), serotonin (70), and acetylcholine (35), most of which shown to be involved in neuromuscular function and molecular and functional analyses are limited. This includes the lack of knowledge about potential ligands binding to orphan schistosome GPCRs.

In this study, we examined the rhodopsin orphan GPCR *Sm*GPCR20, previously identified in a transcriptomics study, to be differentially transcribed between females and males (25, 37). Phylogenetic analyses revealed a close relationship of *Sm*GPCR20 to orthologs in *S. japonicum, S. haematobium,* and other flatworms, whereas orthologs of the nematode *C. elegans*, the intermediate host snail *B. glabrata*, *Homo sapiens*, and others appear more distantly related. This finding grouped *Sm*GPCR20 into a platyhelminth-associated rhodopsin-like orphan-family (66). The high conservation among schistosome species suggests a more specialized role(s) of *Sm*GPCR20 for Schistosomatidae. Using the MALAR-Y2H system (40), we identified the neuropeptides *Sm*NPP26 and *Sm*NPP40 as potential interaction partners. Expression analyses by qRT-PCRs indicated a male-preferential transcript profile (with sM > bM tendency) for *Smgpcr20* and for both *npp* genes higher mRNA levels in unpaired vs paired worms. At this stage of the analysis, it is not yet clear what these potentially higher transcript levels in unpaired worms (except *Smgpcr*20 in females) sM > bM; sF > bF) mean. It is tempting to speculate that these genes may also contribute to male-female attraction to establish a pairing contact. However, since transcript levels of a gene may not be representative for its protein level after translation, further studies are needed to substantiate the pairing influence on the expression of these three genes. WISH results of *Smgpcr20*, *Smnpp26*, and *Smnpp40* suggested their localization in neuronal cells, an interpretation supported by comparable signal patterns of other neuronal genes in *S. mansoni* (33, 43, 71). In the free-living flatworm *Schmidtea mediterranea*, similar signal patterns were found for neuropeptides involved in regeneration processes of the central nervous system (72). According to single-cell atlas data of *S. mansoni* (43), *Smgpcr20* is preferentially transcribed in the parenchyma but also in some neuronal clusters (1, 2, and 4), and in neoblasts (43). These single-cell data indicated a low expression of *Smnpp26* in all male tissues except neuron cluster 14, which exhibited a high transcript level (Fig. S5C) (43). In paired females, the highest *Smnpp26* transcript levels also occurred in neuron cluster 14. Of note, *Smnpp26* transcript levels differed in females depending on the pairing status. The overall transcript level across the majority of tissues was higher in sF than bF, which corresponded to the RNA-seq data (37) and the qRT-PCR data obtained here. However, while the *Smnpp26* transcript level was high in neuron cluster 4 in bF, it was nearly absent in this cluster in sF (Fig. S5C). In contrast, *Smnpp26* transcript levels were high neuron cluster 14 cells of sF and bF; however, this cluster seemed to be larger in females compared to males (43). In the anterior head region of females, WISH indicated differences of the *Smnpp26* signal patterns with more stained cells in sF compared to bF. Therefore, we hypothesize that these additional cells in sF may belong to neuron cluster 14, while neuron cluster 4 cells occur along the sF body and in bF from the uterus down to the part containing the vitellarium tissue. Some *Smnpp26*-positive cells have similar locations as cells in females and males expressing *Smgpcr20*. Indeed, colocalization experiments by double FISH provided first evidence for *Smgpcr20 and Smnpp26* transcripts in cells positioned in the same subtegumental area of the anterior region as found by WISH in experiments with the individual genes/transcripts. This suggests an autocrine and/or paracrine effect of *Smnpp26* for subtegumental cells.

*Smnpp40* transcript occurrence appeared to differ in females depending on the pairing status with a tendency of higher overall transcript level across the majority of tissues in sF than bF, especially in the anterior part. These differences corresponded to the RNA-seq data (37) and the qRT-PCR data obtained here. The single-cell atlas data indicated high transcript levels in neuronal cells, which dominated in clusters 1, 8, 13, 15, and 21–23 with no obvious difference between sF and bF, except that neuron cluster 1 was found to be enlarged in sF compared to bF (43). Furthermore, there are quantitative differences between females and males, with bM showing higher transcript levels than females in neuron clusters 2, 4, 6, 10, 16, and 30, but lower transcript levels than females in cluster 1. Also in this case, double-FISH results indicated *Smgpcr20* and *Smnpp40* transcripts in cells positioned in the subtegumental area of the head region as found by WISH in experiments with the individual genes/transcripts. The position of *Smnpp40* signals in the anterior part overlapped to some extent with the signals of *Smgpcr20* and *Smnpp26*. Also in this region, autocrine and/or paracrine effect might happen. Beyond that, we detected *Smnpp40* signals around the ootype, where no *Smnpp26* signals occurred. This localization coincided with the WISH signals obtained for *Smgpcr20*. Indeed, colocalization experiments by double FISH indicated *Smgpcr20* and *Smnpp40* transcripts in cells surrounding the ootype, where we found no evidence for *Smnpp26* signals (Fig. S11). This suggests an autocrine and/or paracrine effect of *Smnpp40* for cells surrounding the ootype.

The localization of neuropeptides in somatic gonad tissue, to which the ootype belongs, was also reported for *C. elegans* before (73). Other studies have shown that a neuropeptide of the NPY family member was expressed in the shell glands that are involved in egg encapsulation and deposition in *S. mediterranea* (74, 75). In *Fasciola hepatica*, FMRFamide-immunoreactive cell bodies have been observed among the cells of Mehlis’ gland around the ootype (76). In *S. mansoni*, expression of an NPY-like family member has been detected in the region of Mehlis’ gland (77). However, *Sm*NPP26 and *Sm*NPP40 exhibit no similarity to NPY (with the conserved C-terminal Y_(−17)_Y_(−10)_ GRPRF.NH_2_ motif) (78) and FMRFamide (with C-terminal-RF.NH_2_ motifs) (78) neuropeptides. Thus, the area surrounding the ootype may be equipped with neuronal cells harboring NPY-like and FMRFamide as well as novel NPP families such as *Sm*NPP40 in parasitic flatworms, which have not yet been classified, even though there are similar peptides in free-living flatworms. We hypothesize that *Sm*GPCR20 in combination with *Sm*NPP40 may have a function in regulating egg-forming processes in the ootype by contractions, which can be observed in living schistosomes by microscopy (Grevelding, unpublished). Transport of formed egg occurs via the uterus, which depends on contractions as well to support egg export via the gonopore, a structure forming the end of the uterus of schistosomes (79). Besides the localization, the assumption of contraction influence on egg formation matched the RNAi phenotype observed since *Smgpcr20/Smnpp40* double KD led to a reduced number of eggs compared to the untreated control. The results obtained following *Smgpcr20/Smnpp26* double KD indicated an additional role of this neuropeptide for egg formation, although *Smnpp26* transcripts were not localized around the ootype.

The area around the ootype of schistosomes is filled by the Mehlis’ glands, which are known to support egg-shell formation by releasing secretory granules composed of neutral glycoprotein into the ootype (80, 81). It seems less likely that the WISH signals originated from this tissue because in the single-cell atlas, the list of genes found to be expressed in the Mehlis’ glands does not contain one of the three genes in focus. By indirect immunofluorescence using antisera of pancreatic polypeptides, including NPY immunoreactivity, was demonstrated in nerve cells along the vitelline duct and in the wall of the ootype, but most notably in a cluster of cells in the region of Mehlis’ gland (77, 80). This supports our assumption of the activity of the three genes in neuronal cells that are located in between the Mehlis’ gland around the ootype. With respect to all transcript data available by now, it is tempting to speculate that cells of the neuronal clusters 1 and/or 3, in which both *Smgpcr20* and *Smnpp40* are transcribed (Fig. S5), may represent the WISH-positive cells circularly surrounding the uterus. Antibody-based mapping of neuropeptides and other marker cells for neuronal clusters may solve this and similar questions in the future.

Functional analyses suggested roles for *Smgpcr20, Smnpp26,* and *Smnpp40* in controlling the length and width of the ovary in females, along with an effect on oocyte differentiation and egg production, which was reduced in all RNAi groups. Since mRNA of all three genes was not detected in the reproductive organs in females, this discrepancy can be explained by indirect RNAi effects, which may have downregulated neuronal and/or neuromuscular activities potentially controlled by the three genes. One consequence could be the downregulation of reproduction-associated processes such as the pairing-controlled maintenance of ovary differentiation and oocyte differentiation. Remarkably, a previous study in *Drosophila melanogaster* showed an interorgan communication between the gut and the ovary that promotes mating-induced activation of gametogenesis (82). Since the expression levels of *Smgpcr20* and *Smnpp40* were higher in bM compared to bF (similar levels for *Smnpp26*), it may be possible that *Smgpcr20* interactions with both *npps* may exert a male-dependent function in this context. At the physiological (motility/vitality, pairing capacity *in vitro*) and morphological level (CLSM based), there was no visible phenotype in the male that could explain a preferential role of these three genes for the male itself. This does not exclude potential roles of these genes that have not been covered by our phenotype analyses in males.

As determined by ImageJ analyses of 10 females of each treatment group, *Smgpcr20*/*Smnpp26* double KD and triple KD also led to a significant decrease of the length of first-time paired females compared to control females, which were paired and maintained under the same conditions without dsRNA or with a high amount (60 µg/mL) of irrelevant *ampR* dsRNA. This size reduction effect was not significant after *Smgpcr20*/*Smnpp40* double KD. The ovaries of females of all RNAi groups were also reduced in size, which was more pronounced in the *Smgpcr20*/*Smnpp26* and triple KD groups than in the *Smgpcr20*/*Smnpp40* KD group. Only few mature and immature oocytes were visible in the *Smgpcr20*/*Smnpp26* double KD and triple KD groups. In contrast, the number of immature oocytes in the *Smgpcr20*/*Smnpp40* double KD group was similar to the control, but the number of mature oocytes was also decreased, which suggests a higher influence of the activities of both genes of oocyte maturation than on oogonia division. This finding was comparable to the analogous KD approach with pairing-experienced females.

To get first insights into molecular processes downstream of *Sm*NPP26*/Sm*NPP40-activated *Sm*GPCR20, we investigated the transcript levels of marker genes known to be involved in egg synthesis, oocyte differentiation, and stem-cell activity following RNAi. The egg-shell precursor genes, *Sm*p14 (47) and fs800 (48), and the egg-synthesis genes, *Sm*TYR1 and *Sm*TYR2 (49, 50), were significantly downregulated in the *Smgpcr20*/*Smnpp26* double KD and the triple KD groups. In these two groups, the transcript levels of the germline stem-cell marker *nanos-1* (52) and bF expression-biased genes *Smmyst4* (vitellarium marker) (37) and *Smsod* (redox marker expressed in the vitellarium) (37, 45) also decreased after RNAi, which corresponded to the results of the *Smgpcr20*/*Smnpp26* double KD and triple KD that induced impairment of the gonadal stem-cell proliferation. However, the expression levels of the somatic stem-cell marker *nanos-2* (52) and GSC progeny marker *Smmeiob* (43) were not affected in first-time paired females upon any dsRNA treatment. The expression levels of none of these genes changed significantly in the *Smgpcr20*/*Smnpp40* KD group. These results indicate that the potential ligands *Sm*NPP26 and *Sm*NPP40 may fulfill only in part similar roles in activating *Sm*GPCR20. Although in all RNAi combinations, egg production was affected in pairing-experienced mature females, differences were seen with respect to the ovary phenotypes of these and first-time paired females. We observed clear RNAi phenotypes in the *Smgpcr20*/*Smnpp26* double KD group, which assigns the neuropeptide *Smnpp26* a major role in this context. This involved the growth retardation phenotype discovered in first-time paired females, which was not found to be significant in the *Smgpcr20*/*Smnpp40* double KD group. Roles of neuropeptides for reproduction have also been shown in planarians, in which the neuropeptide receptor NPYR1 of neuroendocrine cells receives signals from NPY8, which occurs in the CNS to regulate germline development including GSC differentiation (26, 27). *Sm*SOD is an important redox protein, which belongs to the Cu-Zn superoxide dismutase family, defending cells against reactive oxygen species by converting the superoxide radical to molecular oxygen and hydrogen peroxide (45, 54). *Smsod* mRNA was detected in the vitellarium of females (45). The observed downregulation of *Smsod* transcripts suggests that the *Sm*GPCR20-*Sm*NPPs interactions could also be involved in the redox system, which is active in the female reproductive system. *Sm*MEIOB has been shown to regulate meiosis progression of GSC progeny (83). Since we found no evidence for differential regulation upon RNAi, *Sm*GPCR20-*Sm*NPP interactions may not be involved in meiosis initiation. Instead, egg production-associated genes were significantly downregulated upon *Smgpcr20*/*Smnpp26* RNAi, which fits to the reduced egg production in this KD group. Although a clear phenotype was not found in the vitellarium, the ovary showed a reduced size, and oocyte differentiation seemed to be affected as well.

In summary, our study provided first evidence for specific interactions between *Sm*GPCR20 and two *Sm*NPPs. The functional analyses by WISH and RNAi suggest a multifaceted role of *Sm*GPCR20 in concert with *Sm*NPP26 and *Sm*NPP40 for the growth of first-time paired females, oogenesis, and egg production. Thus, our results contribute to the understanding of rhodopsin-family GPCRs, their potential ligands, and their roles for the biology of *S. mansoni*. Further studies are needed to substantiate the specificity of SmNPP-GPCR20 interaction, e.g., by fluorescence energy transfer (FRET) analysis or related techniques. With respect to their druggability and the urgent need to find alternative treatment options to fight schistosomiasis, unraveling the biological function(s) of GPCRs will lead us to potential targets for the design of novel drugs—especially when receptors are in focus that exhibit a low homology to their host counterparts.

## MATERIALS AND METHODS

### Parasite maintenance

Adult and larval schistosome stages originated from a Liberian isolate of *S. mansoni*, which was maintained in snails (*Biomphalaria glabrata*) and Syrian hamsters (*Mesocricetus auratus*) (84, 85) in accordance with the European Convention for the Protection of Vertebrate Animals used for Experimental and Other Scientific Purposes (ETS No 123; revised Appendix A). Adult worms were obtained by hepatoportal perfusion at 42–49 days post infection (p.i.). In case unisexual worm populations (single sex, ss) were required, snails were infected by a single miracidium (mono-miracidial infection) to obtain clonal cercariae for hamster infection. In this case, perfusion occurred 67 days p.i.. In case of mixed-sex worm populations (bisex, bs), snails were infected by 10–15 miracidia (poly-miracidial infection) to obtain cercariae of both sexes for hamster infection. In this case, perfusion occurred 46 days p.i.. After perfusion, worms were transferred to Petri dishes of 60-mm-diameter size containing 3 mL of M199 medium [Sigma-Aldrich; supplemented with 10% newborn calf serum, 1% HEPES ( 2-(4-(2-yydroxyethyl)-1-piperazinyl)-ethan sulfonic acid; 1 M), and 1% ABAM (antibiotic antimycotic) solution (10,000 units of penicillin, 10 mg of streptomycin, and 25 mg of amphotericin B per milliliter)] in groups of 20 couples per Petri dish until further usage. All worms were cultured *in vitro* at 37°C and 5% CO_2_.

### *In silico* analyses

We used InterPro (https://www.ebi.ac.uk/interpro) for protein family identification. MEGA11 software (86) was used to construct the phylogenetic tree based on the maximum likelihood method, which was done using the Bootstrap method with 1,000 bootstrap replications. As bioinformatics tools to predict TM domains for *Sm*GPCR20 and rhodopsin-like orphan GPCRs in *S. japonicum* and *S. haematobium*, we used DeepTMHMM (https://dtu.biolib.com/DeepTMHMM) (87) and SACS HMMTOP (https://www.sacs.ucsf.edu/cgi-bin/hmmtop.py) (88). Multiple sequence alignments were performed with Clustal Omega (https://www.ebi.ac.uk/Tools/msa/clustalo/) (89).

For *Sm*NPP26 and *Sm*NPP40, all gene or protein sequences were extracted from Uniprot website or WormBase ParaSite (90), and we performed the BLASTp searches on NCBI (https://www.ncbi.nlm.nih.gov/) with the NPPs compiled from the literature (41, 78). After collecting all the sequences, multiple sequence alignments were done using Clustal Omega. Cleavage site prediction was supplemented by the ProP1.0 server (http://www.cbs.dtu.dk/services/ProP/) (91).

### Cloning procedures

The neuropeptide cDNA library was generated as described previously (40), by cutting pGAD SP-WBP1_cloning_linker_TMP_Cub_ GAL4 with *Acc65I* and *SmaI*, and by ligating annealed primers with corresponding 3′ and 5′ overhangs (Table S1).

Prey plasmids were created by digesting pGBKT7 OST1-NubG with the restriction enzymes *NcoI* and *NotI* and assembly with a gBlock utilizing Gibson Assembly. The resulting vector pGBKT7_SP_OST1_cloning_NubG was modified as described previously (39). The CDS of GPCR20 was RT-PCR amplified using Q5 polymerase using cDNA obtained from a total RNA preparation of *S. mansoni*. Primers were designed to assemble products into pGBKT7_SP_OST1_cloning_NubG digested with *NcoI* and *SmaI* (Table S2).

For the synthesis of double-stranded RNA, we cloned appropriate constructs in pJC53.2 (pJC53.2 was a kind gift from Jim Collins; Addgene plasmid #26536; http://n2t.net/addgene: 26536; RRID: Addgene _26536) (26). To this end, cDNA fragments of *Sm*GPCR20, *Sm*NPP26, and *Sm*NPP40 were amplified by specific primers (Table S3) using AccuPrime *Taq* DNA Polymerase, High Fidelity Kit (Invitrogen, 12346086). The cDNA was obtained from total RNA preparation of *S. mansoni*. RNA was isolated with the Monarch Total RNA Miniprep Kit (NEB, T2010S), RNA quality and quantity were checked using the BioAnalyzer 2100 (Agilent Technologies, USA). A total of 200 ng RNA was reverse transcribed into cDNA using the QuantiTect Reverse Transcription Kit (Qiagen), and cDNAs were diluted 1:7–1:10 in RNase-free water. Both steps were carried out following the manufacturer’s instructions. Amplified PCR products were resolved by agarose gel electrophoresis and extracted from agarose gels with the Monarch DNA Gel Extraction Kit (NEB, T1020S). Subsequently, purified DNA products were ligated with 50 ng of *AdhI*-digested pJC53.2 by T4 DNA Ligase (NEB, M0202S) in a ratio of 3:1 and used to transform the DH5a strain of *E. coli*. All generated constructs were sequenced to prove their integrity (Sanger sequencing; LGC Genomics, Germany).

### Yeast two-hybrid assays

*Saccharomyces cerevisiae* strain Y187 was transformed with the bait plasmid expressing *Sm*GPCR20 and mated overnight with the AH109 strain transformed with plasmids expressing ligand fusion proteins (NPP), as described before (39, 40). The chemokine CXCL12 and its known receptor CXCR4 served as positive control. OST1 is a transmembrane protein of *S. cerevisiae* and, in combination with CXCL12, used as the negative control. After incubation, diploid yeast cells were plated on selective medium lacking -Leu/-Trp and then on selective medium lacking -Leu/-Trp/-His/-Ade. The plates were incubated at 30°C in an incubator.

### RNAi analysis

dsRNA was generated as previously described (26). Briefly, cDNA was amplified by PCR from recombinant pJC53.2 plasmids with Q5 High-Fidelity DNA Polymerase (40 U/µL, NEB, M0491S) and specific primers (Table S3), and the products confirmed by agarose gel electrophoresis. dsRNA was synthesized by *in vitro* transcription with T7 RNA polymerases as follows: 5 µL PCR product (5 µg), 10 µL 10× reaction buffer, 20 µL 25 mM rNTP (NEB, N0450S), 5 µL T7 RNA polymerase (self-made), 1 µL pyrophosphatase, inorganic (IPP) (NEB, M0361), and DEPC (diethyl dicarbonate) water were combined to a final volume of 100 µL. The reaction was incubated for 4 h or overnight at 37°C. To remove any residual DNA, 5 µL of RNase-free DNase I (2 U/µL, NEB, M0303) was added and the mix incubated for 30 min at 37°C; dsRNA was stored at −20°C until use.

For RNAi analysis, 10 couples each were cultured in 3 mL M199 3+ medium at 37°C and 5% CO_2_ in 6-well cell-culture plates (Greiner, Germany) in the presence or absence (control) of dsRNA (60 µg/mL in total) (three technical replicates each). The experimental groups contained worm pairs treated with dsRNA combinations of *Sm*GPCR20 and *Sm*NPP26, *Sm*GPCR20 and *Sm*NPP40, or a combination of all three specific dsRNAs, respectively. As a negative control, animals were not treated with any dsRNA. All parasites were cultured for 15 days, the culture medium and dsRNA were replaced every second day. RNAi effects were monitored by determining pairing stability, motility, and egg production, which were recorded by bright-field microscopy (Leica, Germany). Egg production was determined by counting eggs every 2 days. After 15 days, 10 worms were harvested for qRT-PCR analysis to determine mRNA levels of *Sm*GPCR20, *Sm*NPP26, and *Sm*NPP40, respectively, and for morphologic examination (five technical replicates each). All experiments were performed in biological triplicates (*n* = 3).

To prepare pairing and RNAi experiments with single-sex females, sF and bM were kept separately for 1 week in culture. During this period, 20 sF and 30 bM were treated with 15–30 μg/mL dsRNAs of target gene combinations (60 µg/mL in total) separately in 6-well cell-culture plates in 5 mL M199 3 medium at 37°C and 5% CO_2_. At day 8, treated sF and bM were combined in a Petri dish (10 sF/15 bM) ratio in fresh ABC169/0.25% low-density lipoprotein (LDL) (16) medium (BM169 was supplemented with 200 µM ascorbic acid (Sigma-Aldrich), 0.2% (vol/vol) human-washed RBCs (10% suspension), and 2.5% human LDL (TRINA, Switzerland). This 1.0/1.5 female/male ratio was shown before to be suitable for 100% pairing efficiency during 48 h (16). Media changes were done every 2 days.

### Confocal laser scanning microscopy

For morphological analyses by CLSM, worms were fixed in AFA (95% ethanol, 3% formaldehyde, and 2% glacial acetic acid) for at least 24 h at 4°C. After staining with Certistain carmine red (Merck, 1390-65-4), as previously described (92, 93), worms were destained in acidic 70% ethanol and dehydrated progressively in 90% and 100% ethanol. Worms were mounted on glass slides with Canada balsam (Sigma-Aldrich, 03984). A TSC SP5 inverse confocal laser scanning microscope (Leica, Germany) was used for imaging. Carmine red was excited using an argon-ion laser at 488 nm. For fluorescence *in situ* hybridization, samples were imaged on an inverse CLSM. Cy3 and Cy5 were excited with 561 and 633 nm, respectively.

For EdU labeling and detection of proliferating cells, the Click-iT Plus EdU Alexa Fluor 488 Imaging Kit (Thermo Fisher Scientific) was used. After 24 h of incubation with EdU, couples were separated, fixed, and stained as described (94). Worms were counterstained with 2′-[4-ethoxyphenyl]-5-[4-methyl-1-piperazinyl]-2,5′-bi-1H-benzimidazole trihydrochloride trihydrate (Hoechst 33342) in a final concentration of 8 µM. Stained worms were examined on an inverse CLSM (Leica TSC SP5; Leica, Germany). Hoechst was excited with a 405-nm laser and Alexafluor488 with an argon-ion laser at 488 nm.

### Quantitative RT-PCR

To assess transcript levels of *Sm*GPCR20, *Sm*NPP26, and *Sm*NPP40, RNA extraction and cDNA synthesis were done as described before. To this end, the cDNA was diluted 1:7 in RNase-free water before use in subsequent qRT-PCR analyses. These were performed in a Q-Rex cycler (QIAGEN) in 20 µL reactions. We designed each primer pair (Biolegio; Nijmegen, the Netherlands) for a melting temperature of 60°C and amplicon sizes of 140–200 bp (Primer3Plus) (95) and determined primer efficiencies as described before (55) (Table S3). Reaction conditions were as follows: initial denaturation step at 95°C for 3 min, 45 cycles at 95°C for 10 s, 60°C for 15 s, and 72°C for 20 s; all samples were measured in triplicate. Fold change of gene expression levels between dsRNA-treated worms and control worms (without dsRNA) was calculated using the 2^−∆∆Ct^ method (96). Transcript levels of *Sm*GPCR20, *Sm*NPP26, and *Sm*NPP40 were normalized to the level of *Smletm1* (Smp_065110), a proven reference gene for *in vitro* studies (55). Gene expression levels of bM, sM, bF, and sF were calculated using the formula: relative expression = 2^−∆Ct^ × *f*, with *f* = 1,000 as an arbitrary factor (42).

### Whole-mount *in situ* hybridization

Whole-mount colorimetric *in situ* hybridization and fluorescence *in situ* hybridization were performed as previously described (26). Separated males and females were prepared by incubation in 0.6 M MgCl_2_ for 1 min while shaking and then fixed for 4 h in 4% formaldehyde dissolved in PBSTx (1× PBS, 0.3% Triton X-100) at room temperature. Fixed worms were dehydrated in 100% methanol and stored at −20°C. Rehydrated by incubation in 50% methanol dissolved in PBSTx, samples were bleached for 1 h in bleaching solution (9 mL DEPC H_2_O, 500 µL formamide, 250 µL 20× SSC pH 7, 400 µL 30% H_2_O_2_) under direct light. After bleaching, the samples were rinsed with PBSTx and subsequently treated with proteinase K (20 mg/mL, Ambion, AM2546) solution (bM: 45 µg/mL; bF: 25 µg/mL; sF: 7.5 µg/mL dissolved in PBSTx for 45 min, respectively), and we performed WISH with bM, bF, and sF to localize the transcripts of all three genes. Post fixation, the samples were treated with 4% formaldehyde for 15 min. For hybridization, labeled riboprobes were generated using DTG (digoxigenin)-11-UTP (Jena Bioscience, NU-821-DIGX, Germany) and FITC (fluorescein-12-UTP) (Jena Bioscience, NU-821-FAMX-S, Germany). DIG riboprobes were used for WISH, while DIG and FITC riboprobes were used for FISH. Riboprobes were synthesized by PCR from recombinant pJC 53.2 plasmids by Q5 High-Fidelity DNA Polymerase (40 U/µL, NEB, M0491S) (Table S3). In addition, purified PCR products were used to generate riboprobes by *in vitro* transcription with SP6 or T3 RNA polymerases, as follows: 100–500 ng PCR product, 2 µL 10× transcription buffer (Roche, 11465384001), 1 µL T3 (Roche, 11031163001) or SP6 RNA polymerase (Roche, 11487671001), 2 µL DIG-NTP mix (10 mM ATP, CTP, GTP and 7 mM UTP, 3.5 mM DTG-11-UTP), 0.6 µL Murine RNase inhibitor (40 U/µL, NEB, M0314S), and RNase-free water to a final volume of 20 µL. The reaction mix was incubated for 16 h at 27°C. Subsequently, 1 µL of RNase-free DNase I (2 U/µL, NEB, M0303S) was added, and the mix incubated for 20 min at 37°C. For double FISH, the single-stranded RNA probe for *Sm*GPCR20 was labeled with FITC, while *Sm*NPP26 and *Sm*NPP40 were labeled with DIG. FITC riboprobes were generated as described before (26). Probes were stored at −20°C until use.

For WISH, post-hybridization washes and blocking were done as described earlier. For detection, an anti-DIG-AP (1:2,000, Millipore Sigma, 11093274910) antibody was incubated in colorimetric blocking solution [7.5% heat-inactivated horse serum (Sigma-Aldrich, #H1138) in TNT] overnight at 4°C and developed with nitro-blue tetrazolium (Roche,14799526) and 5-bromo-4-chloro-3′-indolyphosphate (Roche, 13513022). Finally, samples were mounted in 80% glycerol. For double FISH experiments, worms were incubated with anti-FITC-POD (1:1,000, Millipore Sigma, 11426346910) in FISH blocking solution [5% heat-inactivated horse serum (Sigma-Aldrich, MFCD00164115) and 0.5% Western Blocking Reagent (Roche, 11096176001) in Tris NaCl Tween 20 buffer solution, pH 8.0 (TNT)] overnight at 4°C and washed in TNT. For tyramide signal amplification, animals were developed in TSA Plus working solution (TSA Plus Stock Solution 1:50 in 1× Amplification Diluent) (TSA Plus Cyanine 3, Akoya Biosciences, NEL744001KT) for 15–30 min at RT in the dark; 300 µL of TSA Plus working solution was used per well. Following development, worms were washed in TNT. Quenched residual peroxidase activity was blocked with 100 mM sodium azide (Sigma-Aldrich, 26628-22-8) in TNT for 45 min at RT and then washed in TNT. Following residual peroxidase inactivation, animals were washed in TNT and incubated in anti-DIG-POD (1:1,000, Millipore Sigma, 11207733910) in FISH-blocking solution overnight at 4°C. This process was repeated with a different fluorescent-tyramide conjugate (TSA Plus Cyanine 5, Perkin Elmer, NEL745001KT), washed with TNT, and incubated with 0.1 µg/mL Hoechst 33342 overnight at 4°C. Samples were mounted in Mount FlourCare (ROTIMount FluorCare, ROTH, HP19.1) for further analysis.

### Statistical analysis

Data obtained during transcript profiling of *Sm*GPCR20, *Sm*NPP26, and *Sm*NPP40, and following RNAi to determine KD efficiencies were given as mean ± SEM, respectively. Significant differences were determined by *t*-test, one-way ANOVA, two-way ANOVA (GraphPad Prism 7), and Tukey’s test for multiple comparisons (97) and indicated as follows: *P < 0.05; **P < 0.01, ***P  <  0.001, and ****P  <  0.0001.

## Data Availability

The sequences of Smp_084270 (*Sm*GPCR20), Smp_071050 (*Sm*NPP26), and Smp_004710 (*Sm*NPP40) are available on the Uniprot website (https://www.uniprot.org/) under accession numbers A0A3Q0KIK8, G4V7X3 , and G4VD53, respectively, or in WormBase ParaSite (https://parasite.wormbase.org; version 9) (90).

## SUPPLEMENTAL MATERIAL

Supplemental figures and tables spectrum.02193-23-s0001.pdf Figures S1-S11 and Tables S1-S3.

ASM does not own the copyrights to Supplemental Material that may be linked to, or accessed through, an article. The authors have granted ASM a non-exclusive, world-wide license to publish the Supplemental Material files. Please contact the corresponding author directly for reuse.
