# Supplementary material for: Rhodopsin orphan GPCR20 interacts with neuropeptides and directs growth, sexual differentiation, and egg production in female Schistosoma mansoni
Source: Microbiol Spectr. 2023 Dec 4;12(1):e02193-23. doi: 10.1128/spectrum.02193-23 (PMC10783048; doi:10.1128/spectrum.02193-23)
Supplement: Supplemental figures and tables — Figures S1-S11 and Tables S1-S3. [file spectrum.02193-23-s0001.pdf]

Supplementary Figures (pages 1 - 22) and Tables (pages 23 - 27)

Supplementary Figure S1

Sequences and accession numbers used for the phylogenetic analyses of the GPCR Smp_084270

Species include: schistosomes (*Schistosoma japonicum* (A0A4Z2CQX1), *S. mansoni* (Smp_084270 (A0A3Q0KIK8)), *S. haematobium* (A0A095C4R8), and *Trichobilharzia regent* (A0A183WMW7)); other trematodes (*Clonorchis sinensis* (H2KRY6) and *Fasciola gigantica* (A0A504Z2P0)); cestoda (*Echinococcus granulosus* (U6J3B5) and *Taenia asiatica* (A0A0R3W0W9)); monogenea (*Protopolystoma xenopodis* (A0A3S5CMM6)); turbellaria (*Schmidtea mediterranea* (A0A193KU74)); nematodes (*Caenorhabditis elegans* (G5EDR2)); molluscs (*Pomacea canaliculate* (A0A2T7PVN7) and *Biomphalaria glabrata* (A0A2C9LEA4)); arthropods (*Apis mellifera* (Q9NG02) and *Daphnia pulex* (E9G3B6)); amphibians (*Xenopus laevis* (B7ZRQ1)); mammals (*Homo sapiens* (Q14439)).

>tr|A0A3S5CMM6|A0A3S5CMM6_9PLAT G_PROTEIN_RECEP_F1_2 domain-containing protein OS=Protopolystoma xenopodis OX=117903 GN=PXEA_LOCUS26816 PE=4 SV=1

```
MANEDPQWPYLDSPWDLLRDCFHIRKLENEHTDWTACILSRILGVLSAYIFPVIGLLGLLVN
CLTAFIFLRCFRTPTRQMIYLACLAVSDGLTILLFGWLWVFPAKGIPYATDGKTYFFTFYSG
WTECRIHRWAYSFTSCLGNNIFLLTTLDRCMSIYLPLKFSRLPQKRAWQMLLAITLVSGVMM
LPFGISTGLYPTQGNKVICWLTEDQTFLQLYHVLFANAGFLQTVLIIAFNLALLIRLRQNAL
LRKQLTCKFTTGRKEVSASILLLLLSTIVVICALPQTVAYMFAFIYQTALPHDTGSMLTRLA
YNISDIGWNLLFLQYTANFFLYLSRMPNFRLATLRLITCRCGQALQRRLEEHYYSSRPAGKT
QTTNMFQVGSKRALVVRSLTEQVKRCQHGRYSQNGPGRICYQDETMVDVWPASPRFHNGPTS
LPTHSFFSHSPSPAISLPQSSPSPPPSSPHLPPPPSLMRISASPPALSSPSSLPSPSPLMKL
QLRLQLPPLSLTAQSSSSPVPTPQLMKLPRYPPSPPTIGHSLSTYSHSPRSFGGSIDKTQPH
WGNRKTSNSQQHVVTFSPPTSRGTRVGESLPDDEVTRF
```

>tr|A0A3Q0KIK8|A0A3Q0KIK8_SCHMA Rhodopsin-like orphan GPCR, putative OS=Schistosoma mansoni OX=6183 PE=4 SV=1

```
MISMNSSELIFTERPQRIFQDCILLVQNYSYIQELKCLSSKVIGTIVGYLIPIFSIPCIIIN
LFIAINVIMKGKKASRQLIYISGICLSSAIANIMFIWLWQYPSYGLPYTTNGTQFFSFLNIS
ITACRFHRFMYSFSATFMCNMRVCASSDRCLAVWKPIMLRKFRHHYAWYVYGVVIFISALLM
LPFAIEVNWIPTKYGLQCWVQSANVYIQIHHAFLSNLGPVQTMILIIIDITFAFKFRQQLKK
NKTDGIDVTSSKQMHRYLLLFISAASYNVLAATQCTFLLLARLGSVSFIKFESGLAYNISDI
LWYINSLREVLDFVIYHRCFHVFSGITSRISKMFYYTSKKTISVSRDISKISVVSGHNY
```

>tr|A0A193KU74|A0A193KU74_SCHMD GCR027 OS=Schmidtea mediterranea OX=79327 GN=gcr027 PE=2 SV=1

```
MDNGTFPIFNMSFHSRYNWAYVGLFIIPMLSMWGNSLVCISVWVEVGLRKRFNYFLVSLAMS
DFLCAILVMPISAWKMIDNYYLETINHQWCLIWYSLDVFFTASTIIHLCTISIDRYNALKNP
LKIHGQKRYRSLVVQLVCAWIIPFSIACPLFLFALELDKTREHSSFKGCGPQNAYFILIATI
```

VTFFIPLIIMTVTYVLTVKILYVQRKEAEANLYSNAVASLVFKPKLSSSLAKFTRTPSTKVM
SEKKLNGKNENSSAMHRRSISTSCLVNSLDSEFKETENHNHFEMTPLLAKTTRNNFVENTSM
NLCGSDNLKPPLQEDQYKQTLPQRQVSFREQQKTKNHYWKSNTVCTVLENPLALLSHQRSFK
MSNKTKTDEYSAFYTKKKETEKQLKQINRSRKAVQTLGILFLLFVICYLPFFVAYLVDFFFK
ICSVSSTAEKMLTFLEWMGYSGSMFNPIVYHFFNPIFRHTYQRLMKCQCYRVFIRHASSSQR

>tr|A0A095C4R8|A0A095C4R8_SCHHA Putative rhodopsin-like orphan GPCR OS=Schistosoma haematobium OX=6185 GN=MS3_0016311 PE=4 SV=1

MNSSELIFIERPQRIFQDCMLLVQNYTYIQELKCLSSKTIGTIVGYLIPIFSIPCIMINLFI
AINVMMKGKKASRQLIYIAGICLSSAIANIMFIWLWQYPSYGLPYTTNGTKFFSFLNISITS
CRFHRFMYSFSATLMCNMRVCASFDRCLAIWKPIKLRKFRHHYAWYVYGVVIFISAVLMIPF
ATEVNWIPTKYGLQCWVLSTNVYIQIHHAFLSNLGPVQTMILIVIDITFAFKFRQQLKKHRT
DGIDVTSSKQMHRYLLLFISAVSYNILAATQCIFLLLARLGTVSFIKFESGLAYNISDILWY
LNSLREVLDFVIYHRCFHVFSGITSRISKMFPYKSKETISISRDISKISIASAYNY

>tr|A0A4Z2CQX1|A0A4Z2CQX1_SCHJA Rhodopsin-like orphan GPCR OS=Schistosoma japonicum OX=6182 GN=EWB00_008264 PE=4 SV=1

MNSSELIFSERPQRIFQECLLLEQNDSYMQQLKCLSSRIIGTLVGYFIPVVSIPCITINLFI
AIIVIINAKKASRQLIYISGICMSSGLANIIFTWLWQYPSYGLPYITNGAKFFSFLNISPEA
CRFHRFMYSFSATFMCNMRVCASVDRCLAIWKPLLLRKFRPHYAWYVYGAFIVISALLMLPF
ATEVNWIPSRYGLQCWVKFTDVYIQIHHAFLSNLGPIQTTLLIIIDITFAIKFRQQLKKHKS
DKIDTTYSKLMRRYLLLFLSAITYNILAATQCTFLLLARLSSVGLIKFDSGLAYNISDILWY
LNSFREILDFIIYHKCFHVFHIITSRLSKAFVNIKNSVSTSRSLSTTTVGSVNNY

>tr|A0A183WMW7|A0A183WMW7_TRIRE G_PROTEIN_RECEP_F1_2 domain-containing protein OS=Trichobilharzia regenti OX=157069 GN=TRE_LOCUS13470 PE=4 SV=1

MNSQEINFAETPKNILFQCQLLKQNYSYIQNLRCLSSSVIGVIVGYLLPVCSIPCILTNLFI
AITVMFKWRTAARQLIYISGICLSSATADVFFIWLWQYPAYGLPYTTNGAKFFTFLNVSPTA
CRFHRFVYSSSATLMCNMRICSSLDRCLAIYVPIKLKKFHHKYAWFVYGATACFSALLMLPL
ATEMDWIPSKYGVQCWFRNANTHPNANNSTNIYLQLHHAFLSNLGPVQTILLIIIDLAFVVK
FRRHLNKHKRHNTTTTDTKKETKTFNRYRLLFISAVSYTLLATVQCIFLALARLSSVEIISF
ETTLAYNMSDILWYLNILREVLDFVIYQKCFRIFNIIVVKVSVICCRPTLSTRSGNLFTRTE
MTTE

>sp|Q14439|GP176_HUMAN G-protein coupled receptor 176 OS=Homo sapiens OX=9606 GN=GPR176 PE=2 SV=1

MGHNGSWISPNASEPHNASGAEAAGVNRSALGEFGEAQLYRQFTTTVQVVIFIGSLLGNFMV
LWSTCRTTVFKSVTNRFIKNLACSGICASLVCVPFDIILSTSPHCCWWIYTMLFCKVVKFLH
KVFCSVTILSFPAIALDRYYSVLYPLERKISDAKSRELVMYIWAHAVVASVPVFAVTNVADI
YATSTCTEVWSNSLGHLVYVLVYNITTVIVPVVVVFLFLILIRRALSASQKKKVIIAALRTP
QNTISIPYASQREAELHATLLSMVMVFILCSVPYATLVVYQTVLNVPDTSVFLLLTAVWLPK
VSLLANPVLFLTVNKSVRKCLIGTLVQLHHRYSRRNVVSTGSGMAEASLEPSIRSGSQLLEM
FHIGQQQIFKPTEDEEESEAKYIGSADFQAKEIFSTCLEGEQGPQFAPSAPPLSTVDSVSQV
APAAPVEPETFPDKYSLQFGFGPFELPPQWLSETRNSKKRLLPPLGNTPEELIQTKVPKVGR
VERKMSRNNKVSIFPKVDS

>tr|U6J3B5|U6J3B5_ECHGR Rhodopsin orphan GPCR OS=Echinococcus granulosus OX=6210 GN=EGR_02845 PE=4 SV=1

MLFAENSRSILPEDPNWPYLDSPWDLLRDCQDYNSDAPNASTLLSACVLSHMLGFVSAYIFP
IVGLFGVVSNLFITYIFLFVFRKPSRQMIYLACVAAADVITIILFGWIWMFPAKGLPYATGA
RVYFFIFNVNNYSCKVMRYLYSFSSCLSSSLFLLTAFDRCLCIYFPLKFARISRRRAWEAVG
VITLFSALVMLPFGLLVEHGFSNGKIICWVQVEPNTLQIYHVLLANSSLLQTVLVIIINIAL
LIRLRQSAVLRETMSKCTSANREIAASMLLVILSTIVVICALPQSIAYIFSTILSQMLNGEA
GRMAVRIAYNISDLGWQLLFFQQASNWVLYMKRMKNFRRATLRLLRCHCSMGRGMVDDAFGS
IHYLSTKARNLTSELRFTRADASKVNGIRFPTTKKGGISDLWKMKYSGGVIGTSFGSSRHQD
RRGDNSNYNISEICRYTYRGSMRHTTTMSTITPGTVYADVDTPTKPTPDPGRSFTRF

>tr|G5EDR2|G5EDR2_CAEEL G_PROTEIN_RECEP_F1_2 domain-containing protein OS=Caenorhabditis elegans OX=6239 GN=srw-33 PE=4 SV=1

MNSSNDLYPGFSDEDIKFWTEIDELLAVLSILFNMLSFLISIIGILPTIFHIIVLSRKSMRT
LTINAFLLGIGICDLARMMFIIMVLGPLYTEHFSHLEHPECMSPNYYSTILFALISGFTGKL
VEYLSIWLAVAMAIIRSLVIKYPLNSRISDLIESKYGIRVLFLITLPIIILSIPKYFRYSIQ
PFGSLWVPPQNCTDFPKNYFQIQYTYVETHIFEKSTDFLTGMEGVLYVIIPSILLPISTLIL
IFQLRISRKKSEALRHTSNSGGDRTTKLVTFMTISFTISTAPFGILHLVKVIVSEAIGSEGM
NLIVDRIASLFPLIITINGAIHFFLCYFLSSQYRDAVREMFGRNKKSKNSISLQHPAMSTVS
TFVKVE

>tr|A0A504Z2P0|A0A504Z2P0_FASGI G_PROTEIN_RECEP_F1_2 domain-containing protein OS=Fasciola gigantica OX=46835 GN=FGIG_07041 PE=4 SV=1

MWHFVDSPLDLIWQCRALEEGGNNVTKVDCVMSTVLGITSAYILPFICGFSLVGTVFFMTVV
VVTKNLISRQFIYLFCMFASNAATSVLFGWLWIFLAKGLPFATNGRVYFFTFYSSPTACSVH
RFAYSFTSTLSCNVLLVASVDRLLCVYFPMEFSNIPKRYGWYVIVITVIVSVFLLVPMAGLM
IWTSVGDKIICWFPDKYQYMEYYHTLISNGGVIQPLAIFVINVIFFVRVRKYAQQLGRVEVL
NAQAKHNIQACVTLLIFSLIFVICALPQSIAYICAYTIIRTNPALTTQIRLAYNVADLFWNL
YFIRDVIYLILMFRLTGICRWFFQFLRGKKHRNKFIISGTFWGQDQMIIVE

>tr|B7ZRQ1|B7ZRQ1_XENLA G_PROTEIN_RECEP_F1_2 domain-containing protein OS=Xenopus laevis OX=8355 PE=2 SV=1

MGDGWGPMECRNRSGTPTTVPSPMHPLPELTHQWTVGMTMFMAAIILLIVMGNIMVIVAIGR
NQRLQTLTNVFITSLACADLIMGLFVVPLGATLVVSGKWLYGSIFCEFWTSVDVLCVTASIE
TLCVISIDRYIAITSPFRYQSLLTKGRAKGIVCSVWGISALVSFLPIMMHWWRDTGDPLAMK
CYEDPGCCDFVTNRAYAIASSIISFYVPLIVMIFVYIRVFKEAQKQMKKIDKCEGRFSHSHV
LSHGRSSRRILSKILVAKEQKALKTLGIIMGTFTLCWLPFFLANVVNVCYRNLIPDKLFLFL
NWLGYANSAFNPIIYCRSPDFRKAFKRLLCCPKKADWHLQTTGELSRTSGGFVNSLDTNALG
TCSECNGVRTSLD

>tr|A0A0R3W0W9|A0A0R3W0W9_TAEAS G_PROTEIN_RECEP_F1_2 domain-containing protein OS=Taenia asiatica OX=60517 GN=TASK_LOCUS3344 PE=4 SV=1

MRFVGSSRTILPEDPNWPYLDSPWDLLRDCHDYNSDAYNTSSHLSACILSHMLGFVSAYIFP
FVGLFGVVSNIFVTYIFLFVFRKPSRQMIYLACVAAADVITIVLFGWIWMFPAKGLPYATGA
RVYFFILNVNTYSCKIMRYLYSFSSCLSSSLFLLTAFDRCLCVYFPLKFARISRRRAWEAVG
VITLFSALSMLPFGVLVGHGLSNGKIICWVQVGPNALQIYHVLLANSSLLQTILVIIINIAL
LIRLRQSAVLRETMSKCTSANREIAASMLLVILSTIVVICTLPQSIAYIFSTILAQVLDGDT
GRTAVRIAYNISDLGWQLLFIQQASNWALYMKRMKNFRRATLRLLRCHCNTGRGWVDDAFGS

LHYLSTKARNFTSELRFTRADTSKTYGIRFPTTKKSGVSDFWKMKYSGGVIRTSFGNFRYQG
KSGNNSINNKSEVCRYTYRGSMRHTTTMSTITPATVYADIDTPTEPALESGRVLTRF

>tr|H2KRY6|H2KRY6_CLOSI Rhodopsin-like orphan GPCR OS=Clonorchis sinensis OX=79923 GN=CLF_107485 PE=4 SV=1

MSRYLELQKNYSFYDTPYDMLVQCEEVRQGLPGASMANCVSSTILGIYSGYILPFVCTFGFL
ANVWTAVLFLLGFRRQTRQLVYLAFLAIANAAIHVLWGWLWLFPAKGLPFMTGARVYYFTFS
QSQEACRLHRFAYSFGSTLSANLLLLAASDRFMCTYWPTKMLRFQRRHAYYAVIAVTVLSIV
MMLPLGICIEWVTIGEKVWCWIDSVYTGVDVYHALFSNACVIQPLCTGVLNICFLVKIRQLL
HKRAQLSSSIGSSGRREVAASETLLVITLVTLCTALPQSAAYNAAFVLSRRPNTQEQTSLAF
NISDIMWCVMLTQTACNIIVYLVRMSAFRKMSLSVIKCQGFRRRVIEGTKDQDKTLQVNMTT
QRSNTENMTTAGAATFRIGSD

>tr|A0A2T7PVN7|A0A2T7PVN7_POMCA G_PROTEIN_RECEP_F1_2 domain-containing protein OS=Pomacea canaliculata OX=400727 GN=C0Q70_00037 PE=3 SV=1

MTTALATTTEMVSNTTHIYDTYDLFIHPHWKQFPLIPEAWHYAIGVYITIVGISGVFGNLLV
IYIFGTTKSLRTPSNMFIVNLALSDLTFSAVNGFPLLTISAFNKRWFFGKVACEFYGLIGGI
FGLMSIDTMAVIAIDRYNVIARPLKASRSLGYRKAFIMIVMVWVWSLIWTLPPLFGWGAYIP
EGFQTSCTFDYLTRTDYFRSYIMCLYICGFAMPLGIIMFCYFFIYRAVAKHEKEMGKMAKKL
NAEIRQGAAAQGSEIKTAKIAMTIITTYLISWLPYATIALIAQFGPAEYVTPYLSELPVMFA
KASAMHNPIIYALSHPKFREVLDTRFPWLLCCCRFSQKEKDAAANTKSQMTRADSVNSNVVG
GNYSNMSR

>tr|A0A2C9LEA4|A0A2C9LEA4_BIOGL G_PROTEIN_RECEP_F1_2 domain-containing protein OS=Biomphalaria glabrata OX=6526 GN=106056607 PE=3 SV=1

MTLLQSKAAGGPRSVLTDATTIGSVVTTDMLADNATEGRHFIPLSDAGFTFIACMLGFTFVV
GSFSNGLCLFVFIRNRRLRSPTNVFVMALNLVDFLMCFTGIPMAMTSAWNHKWIWGDAMCDF
EAFLVYFLGMASMYVLMAIAFDRYIAISKPLLGTKITKSIAYVSCVIWAIPPAFGWNEFGLE
GAGISCSVVWENPDPLYMSYIWAIFFFCFIIPLGIMVYSYWGVLATLRNLNKNSVWDMNSRV
ARKNLAIEKKMFKTAVLIVASYWICWMPYTIVSFISAFIGSEIIPPLFATIPPVIAKCQGIF
NPLILVTRHKAFQKAFFATFVVQRKRKQRTDYSMRVIEVRRTDVLSSCVLS

>tr|Q9NG02|Q9NG02_APIME G-protein coupled receptor OS=Apis mellifera OX=7460 GN=tyr1 PE=2 SV=1

MNSSGESGGTMTEDYDMTGCGPPEEETGSNLPVWEAAAASLTLGFLVLATVLGNALVILSVF
TYRPLRIVQNFFIVSLAVADLAVAILVMPFNVAYLLLGKWIFGIHLCKLWLTCDVLCCTASI
LNLCAIALDRYWAITDPINYAQKRTLKRVLATIAGVWILSGAISSPPLAGWNDWPEELEPGT
PCQLTRRQGYVIYSSLGSFFIPLLLMSLVYLEIYLATRRRLRERARQSRINAVQSTRHREAD
DAEESVSSETNHNERSTPRSHAKPSLIDDEPTEVTIGGGGTTSSRRTTGSRAAATTTTVYQF
IEERQRISLSKERRAARTLGVIMGVFVVCWLPFFLMYVIVPFCPDCCPSDRMVYFITWLGYV
NSALNPLIYTIFNLDYRRAFRRLLRIR

>tr|E9G3B6|E9G3B6_DAPPU G_PROTEIN_RECEP_F1_2 domain-containing protein OS=Daphnia pulex OX=6669 GN=DOP-R PE=3 SV=1

MEIVVVPTLLNVTRWDEGNLTSENATVLVEEKDWDDGNPILALVLFSFCLATVLGNALVIAA
VTRERYLHTVTNYFIMSLAVADCLVGSIVMPFSAAVEMQSDRRWLFGRDLCDVWHSFDVLAS
TASILNLCVISMDRYWAITDPFTYPSRMTPKRAACFIALVWVCSSLISFPAIAWWRAVARIH
PLPEHCVFTDDIGYLVFSSTVSFYGPLSVMVFTYYRIYRAAVAQSRSLRLGIKQVVMASTGE
MGKGTGGGSGSGSGGSGSAETVELLTLRIHRGGRVASDNRRCAAAAALLTYQAANRHQLAID

```
PSSTRKLAKIAKERKAAKTLGIVMGVFIACWLPFFVTNLLSAFCQSCIHNPERVVTVVTWLG
WINSGMNPVIYACWSRDFRRAFARILCGCCPRLFHRWKRHSGKSGTNNPMNVSSRVDQIFIA
TSKFGD
```

>sp|P35359|OPSD_DANRE Rhodopsin OS=Danio rerio OX=7955 GN=rho PE=1 SV=2

```
MNGTEGPAFYVPMSNATGVVRSPYEYPQYYLVAPWAYGLLAAYMFFLIITGFPVNFLTLYVT
IEHKKLRTPLNYILLNLAIADLFMVFGGFTTTMYTSLHGYFVFGRLGCNLEGFFATLGGEMG
LWSLVVLAIERWMVVCKPVSNFRFGENHAIMGVAFTWVMACSCAVPPLVGWSRYIPEGMQCS
CGVDYYTRTPGVNNESFVIYMFIVHFFIPLIVIFFCYGRLVCTVKEAAAQQQESETTQRAER
EVTRMVIIMVIAFLICWLPYAGVAWYIFTHQGSEFGPVFMTLPAFFAKTSAVYNPCIYICMN
KQFRHCMITTLCCGKNPFEEEEGASTTASKTEASSVSSSSVSPA
```

Supplementary Figure S2

Multiple sequences alignment of Smp_071050 (*Sm*NPP26) and Smp_004710 (*Sm*NPP40) orthologs

A

Smp_071050.1_*Sm*NPP26

```
MKIYKFDNKMIIEQLFYCLLVFITFSSIVNAIPVHELNYVNHKTNDPIFGRRYEGRYPTDGNSRFIESE
YSNFYPMAFKRTLFNPILFKRNFDPILFKRSYFDPIIYKRSYFDPILFKRNEDRQFEKREHFDPIIY
```

```
Smed              -MQFRF-----SKMTHV-TLLITVGLFH-----------------VISGYPQTYE-----   31
EgrG              ----------MRAM--LAVLLCALSFFGCVSM--AH-------------PLSDEES---   29
Csin              ---------MMQKNAARICTLCLVLLYLVEASQNMEEKSMDEQHEMFSAYPLSPHESPDI   51
Sjap              MKTYKL---NEKKMFIEQCFYLLLGLLIFSSTVNA--VTVREPN-------YENHGILGN   48
Sman              MKIYKFD----NKMIIEQLFYCLLVFITFSSIVNA--IPVHELN-------YVNHKTNDP   47
Shae              MKIYKFDHNNNNKMIIEQLFYCLLVLMAFSSIINA--IPVHELN-------YVNHKSNDH   51
                                         : :                            .

Smed              -LNQSDRGYYPILFDKRFDPIQFGKRFDPIQFGK--RFDPIQFGK--RFDPIQFGKR-FD   85
EgrG              ------------------LE-LFKRYFDPIRFAMG----PIR------------------   48
Csin              LRQVSEDEHELMD-----SD-KRASHFDPIMFKRLSHFDPIMFKRRAHFDPIMFKRR---   102
Sjap              RLGKHFERQYFVESDY---P-DKRSYFDPIAFKRT-YFDPIAFKR--NFDRILFKRN---   98
Sman              IFGRRYEGRYPTDGNSRFIE-SEYSNFYPMAFKRT-LFNPILFKR--NFDPILFKRSYFD   103
Shae              MIGKQYDERYPTDGNTRFVE-SDYSNFYPMAFKRT-LFNPILMKR--NFDPIVFKRSYFD   107
                                            * *: *       **

Smed              PIQFG-KRFDPIQFGK--RFDPIQFGKRFDPIQFGKKFDPIQFGK--RFDPIMFGR--   136
EgrG              ------------------------TKED-------LDKREAVVASKRYFDPILFKHAY   75
Csin              ------AHFDPIMFKRRAHFDPIMFKRR-------AHFDPIMFKRRAYFDPIMF----   143
Sjap              --------FDPILFKR-SYFDPIAFKRN-------AD---HQFDKREYFDPIIY----   133
Sman              PI----------IYKR-SYFDPILFKRN-------ED---RQFEKREHFDPIIY----   136
Shae              PIIYKRSYFDPIIYKR-SYFDPILFKRN-------EN---RQLEKREYFDPIIY----   150
                                            .         .     .     ****::
```

B

Smp_004710.1_*Sm*NPP40

MTNYWFQLFCCMIIGFLILTSHTNCMDSDNDPSDISAD*KR*FLLALPSP*KR*LSQPSYSFNRY*RR*PMYGYGYRPDYIDADDDLFNED*KR*FLLGLPPKVEH*KR*FLLGLPPSLRQH*KR*FILGLPAPTRFHS

```
Oviv        MPTSKYSKFKKRFALYQSYPNMTYNLYLSCIVTVCFLILTCKTDPLGVEKEWGPNSYSAE  60
EgrG        ------------------------------------------------------------  0
Sjap        --------------------MIVKWSQFIYYITVGLLIFINNTNCIENHL-----SSDDD  35
Sman        --------------------MTNYWFQLFCCMIIGFLILTSHTNCMD---------SDND  31
Shae        --------------------MINYWFQPFCCMIVGFLILTSYINCMD---------SNDD  31

Oviv        LEDADLEKRFLLSLPRPRQRRFFLGLPVMKNRARRVGKRTDEPDYDEYDA--ERGGPLSF  118
EgrG        ----------MILIAS-------T------GAYPHWSVYDSLDTYDDYFDD-FDQQYAQL  36
Sjap        STDMSASKRFLLALPSPKRLSLPS---RLFSRYRRLMAANGYRPYSDYVDNDEEDNIFNE  92
Sman        PSDISADKRFLLALPSPKRLSQPS---YSFNRYRRPMYGYGYRP--DYID--ADDDLFNE  84
Shae        PSDISADKRFLLALPTPKRLSQSS---YTFNRYRRPMYGYGYRP--DYID--SDEDLVNE  84
                   :: :             .   :    .            :*        .

Oviv        RKRFLLGLGSRRHP--------------------------------    132
EgrG        QKRFLLGLGLTKNTPGKEIFFTSVGPKKPNHRQ-------------    69
Sjap        DKRFLLGLPPKAEY---KRFLLGLPPSR-HQKRFILGLPAPTRFQF    134
Sman        DKRFLLGLPPKVEH---KRFLLGLPPSLRQHKRFILGLPAPTRFHS    127
Shae        DKRFLLGLPPKVEY---KRFLLGLPPSHRQHKRFILGLPAPTRFHS    127
             *******    .
```

Suppl. Fig. S2: Shown are prohormone gene sequences with signal peptides highlighted in yellow, italics, and underlined. Identical amino acid residues are highlighted in black, and similar ones in light grey. The predicted propeptides of *Sm*NPP26 (**A**) and *Sm*NPP40 (**B**) could be processed into segments due to the occurrence of potential prohormone convertase (PPC) sites (K, R, KR, KK, or RR); these sites were highlighted in red; Putative mature neuropeptides are in bold, underlined and in green. **A:** *Smed*, *Schmidtea mediterranea* (*Smed-secreted peptide prohormone-15*); *Sjap*, *Schistosoma japonicum* (EWB00_010141); *Shae*, *S. haematobium* (SHAE1_39540); *Csin*, *Clonorchis sinensis* (Csin106202); *EgrG*, *Echinococcus granulosus* (EgrG_000239700.1). **B:** *Sjap*, *S. japonicum* (EWB00_006876); *Shae*, *S. haematobium* (Shae_MS3_00006162); *EgrG*, *E. granulosus* (EgrG_002016900); *Oviv*, *Opisthorchis viverrini* (T265_10151). *indicate sequence identity; : indicates sequence similarity.

Supplementary Figure S3

RNA seq-based transcript profiles of *Smgpcr20*, *Smnpp26*, and *Smnpp40* in adults and gonads

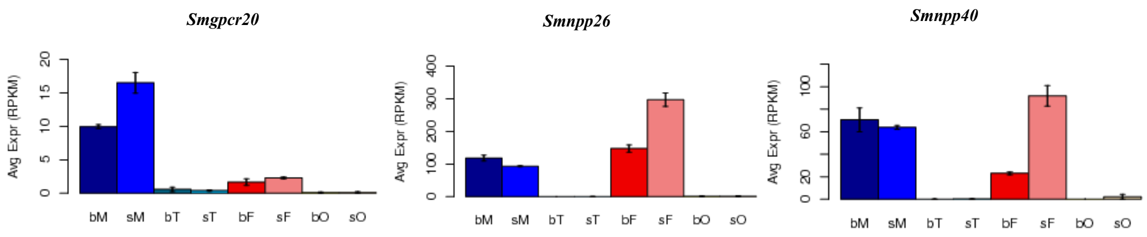

Suppl. Fig. S3: Transcript profiles of *Smgpcr20*, *Smnpp26*, and *Smnpp40* obtained by RNA-seq analysis of female and male *S. mansoni* and their gonads (37). bM, bisex males (pairing-experienced); sM, single-sex males (pairing-unexperienced); bT, testes of bM; sT, testes of sM; bF, bisex females (pairing-experienced); sF, single-sex females (pairing-unexperienced); bO, ovaries from bF; sO, ovaries from sF. Average expression (Avg Expr) was based on RPKM (Reads Per Kilobase per Million mapped reads) values after filtering (37).

Supplementary Figure S4
Cell atlas data

A

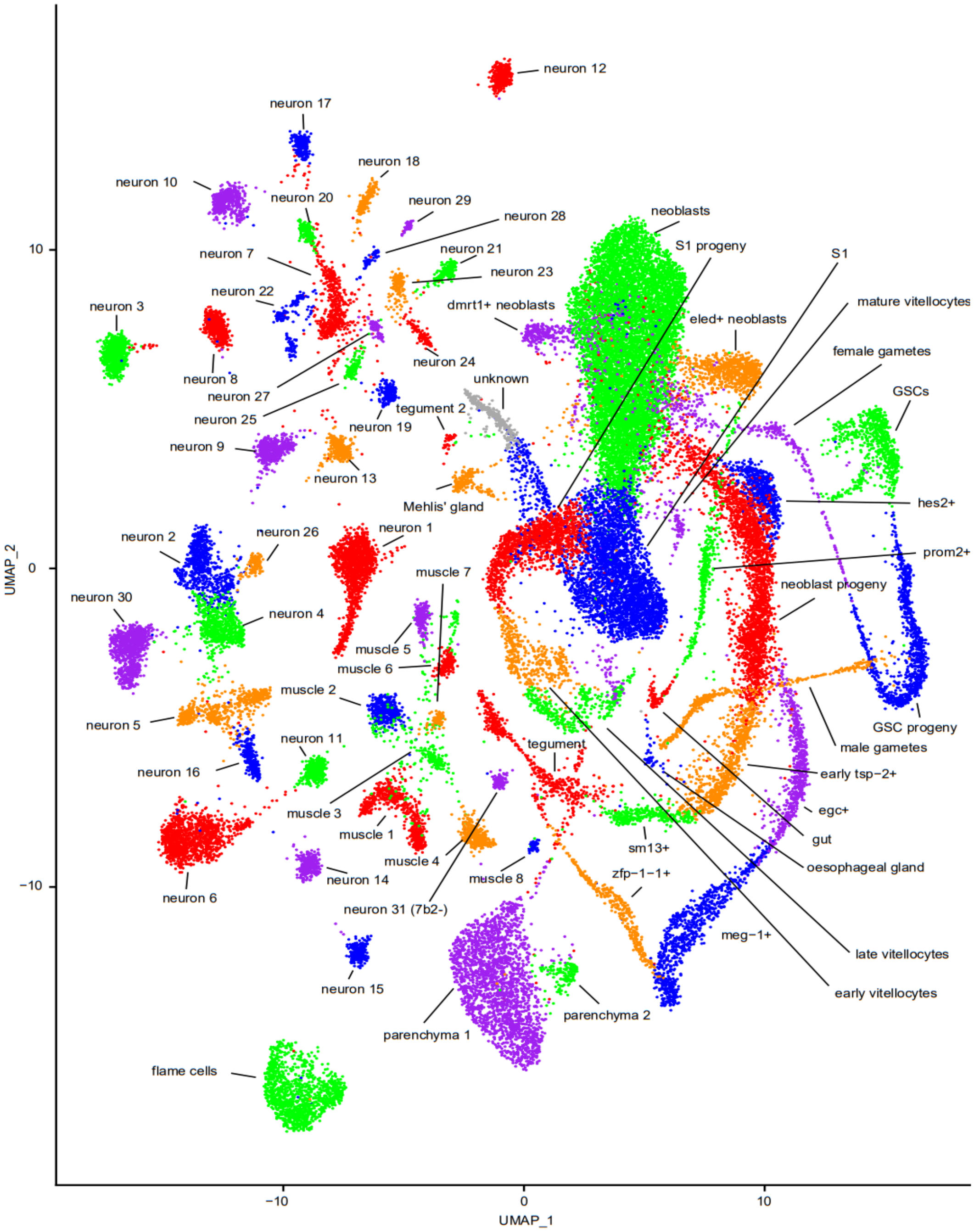

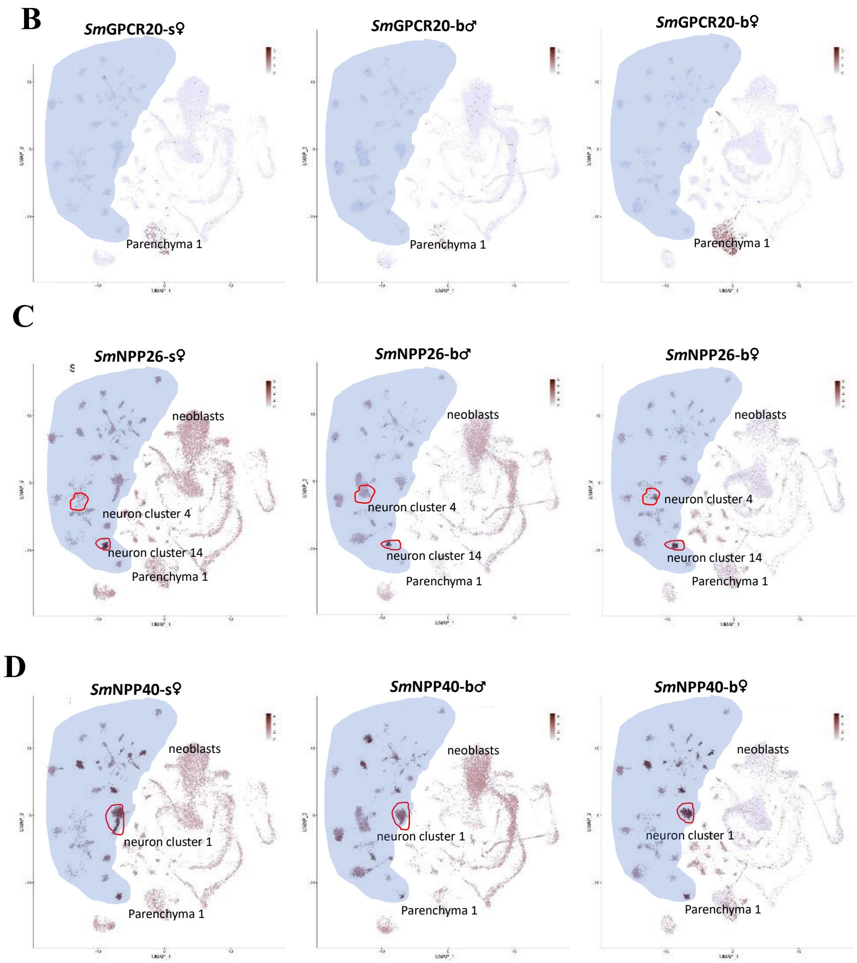

Suppl. Fig. S4: The represented cell atlas data were obtained by single-cell RNAseq analysis (43). (**A**) The overview shows cell clusters assigned to various tissues of *S. mansoni*. (**B**) *Sm*GPCR20 was found to be preferentially transcribed in the parenchyma and weakly in some neuronal clusters - such as 1, 2, and 4. *Sm*NPP26 (**C**) and *Sm*NPP40 (**D**) showed widespead distribution in different cell clusters, especially in neurons and neoblast cells, as indicated. Neuronal clusters are highlighted in light blue.

Supplementary Figure S5

Localization of *Smgpcr20*, *Smnpp26*, and *Smnpp40* in *S. mansoni,* WISH controls

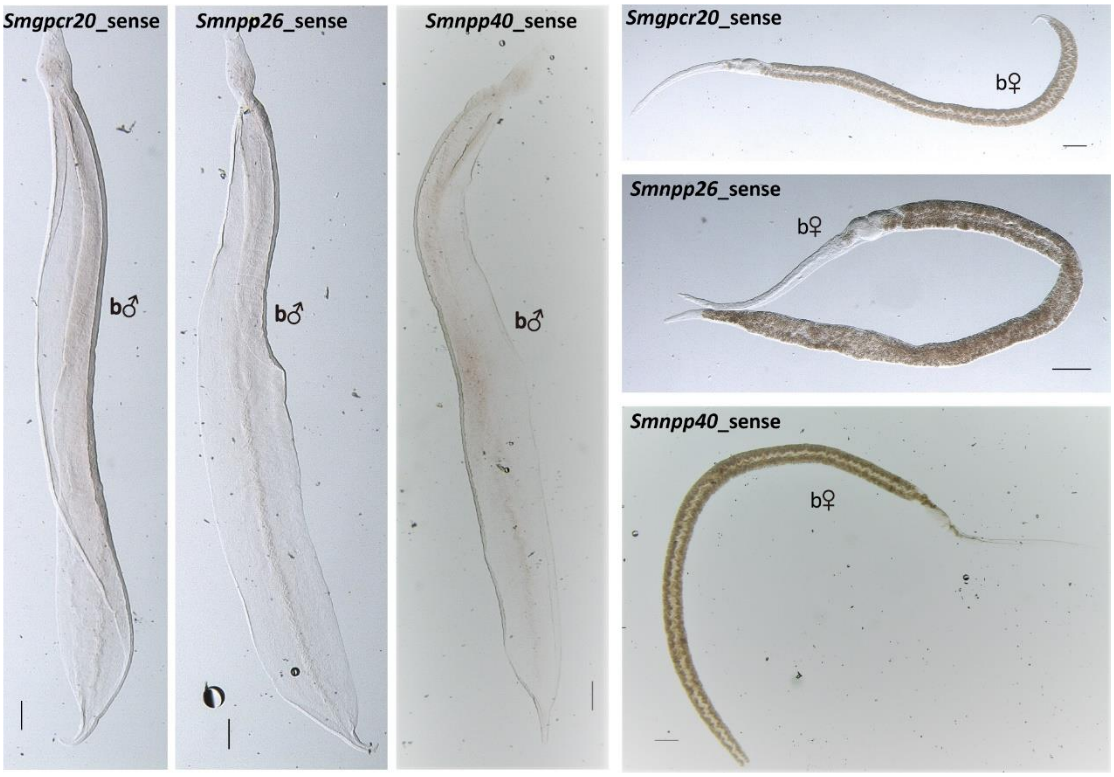

Suppl. Fig. S5: As negative controls for WISH (see Figure 4 and main text), we used sense probes of genes that showed no signals in male and female worms upon hybridization. Scale-bars: 200 µm.

Supplementary Figure S6

Significant reduction of the transcript levels of *Smgpcr20*, *Smnpp26*, and *Smnpp40* upon dsRNA treatment

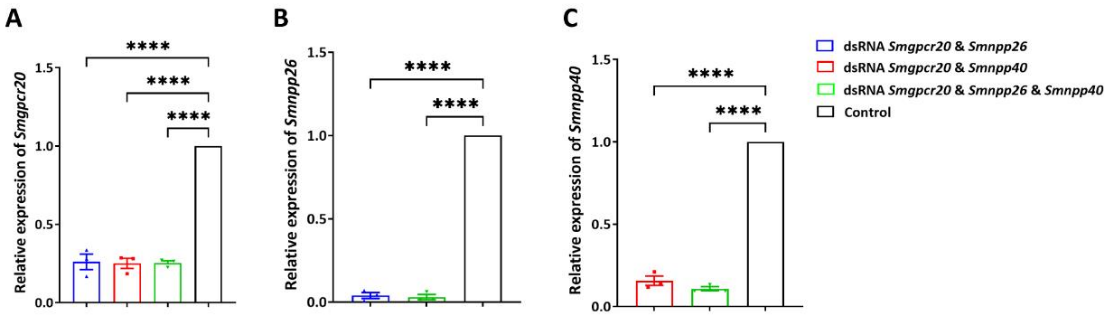

Suppl. Fig. S6. Shown are the relative transcript levels of *Smgpcr20* (**A**), *Smnpp26* (**B**), and *Smnpp40* (**C**), respectively, in *S. mansoni* couples after treatment dsRNA combinations of *Smgpcr20* and *Smnpp26* (blue bar graph), *Smgpcr20* and *Smnpp40* (red bar graph) dsRNA, or a combination of three specific dsRNAs (green bar graph); couples kept under the same conditions but without dsRNA treatment served as control (black bar graph). All dsRNA-treated groups were compared with control (non-treated) groups. Fold changes of gene expression levels between dsRNA-treated worms and the control were calculated using the $2^{-\Delta\Delta Ct}$ method (96). Data are representatives of the mean ± SEM of three separate experiments (n=3). Significant differences were determined by One-way ANOVA and indicated as: $^{****}P < 0.0001$.

Supplementary Figure S7

Physiological assays following RNAi against *Smgpcr20, Smnpp26*, and *Smnpp40*

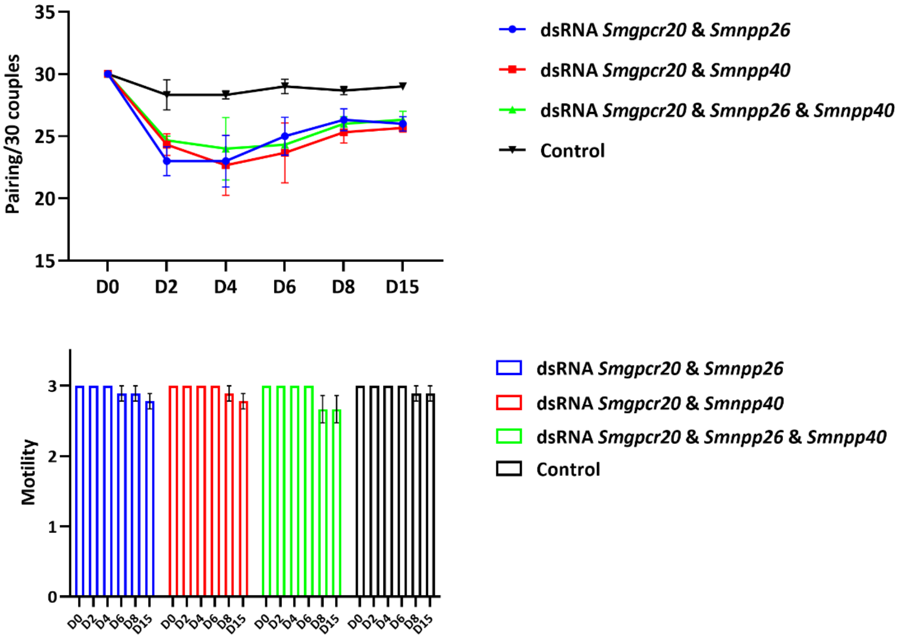

Suppl. Fig. S7: Summary of pairing stability and motility analyses of *S. mansoni* couples *in vitro* following RNAi with dsRNA combinations of *Smgpcr20* and *Smnpp26*, *Smgpcr20* and *Smnpp40* dsRNA, or a combination of three specific dsRNAs. Untreated worms (no dsRNA addition) were used as controls (n = 3; each n with 30 worms). No statistically significant differences were observed. All worms maintained *in vitro* were kept under the same conditions for 15 d.

Supplementary Figure S8

Treatment of *S. mansoni* couples with 60 µg/ml *ampR* dsRNA as further negative control showed no influence on KD efficiencies of the target genes (A), pairing stability (B), egg production (C, D), stem-cell proliferation (E), the size of the ovary (F), and the length of first-time paired females (G).

A

KD was determined separately for males and females after separating couples after 15 d dsRNA treatment

***Smgpcr* 20**

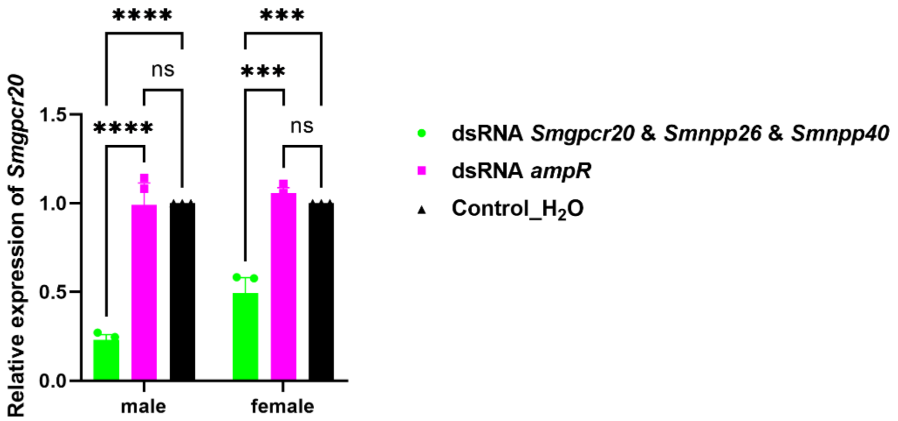

***Smnpp* 26**

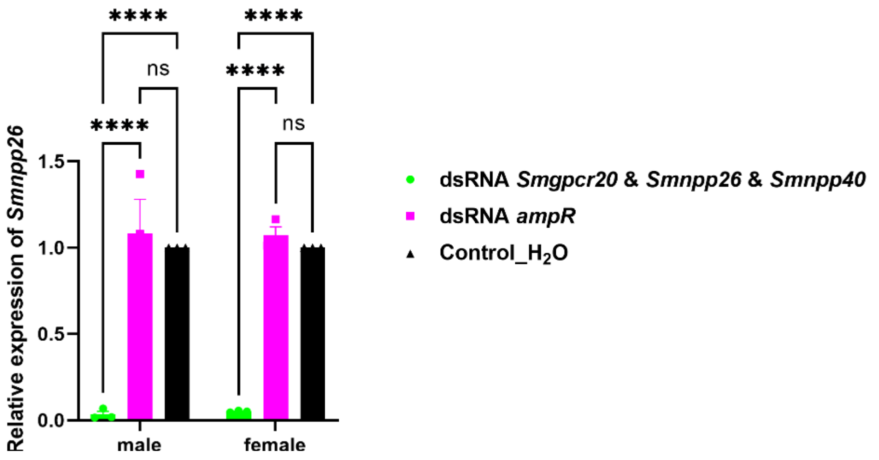

Smnpp40

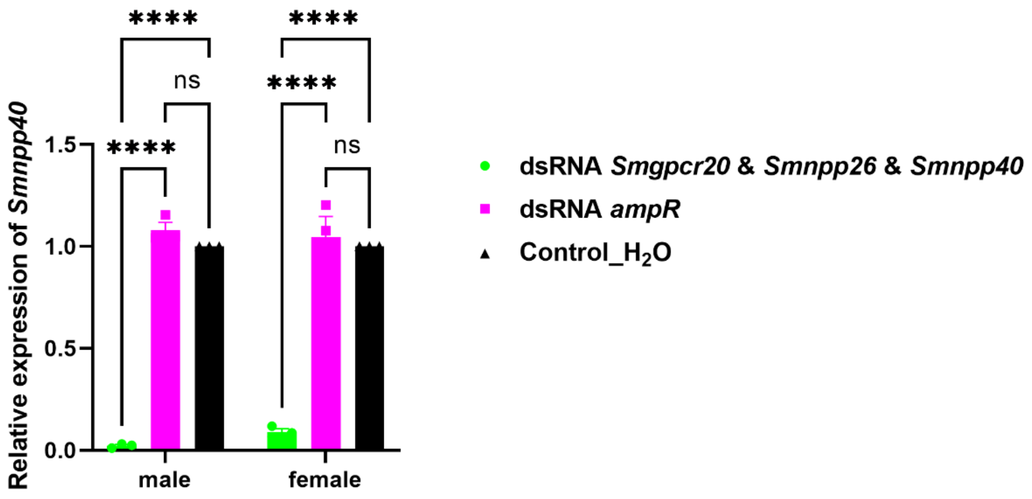

B

None of the different dsRNA treatments caused a significant effect on pairing stability

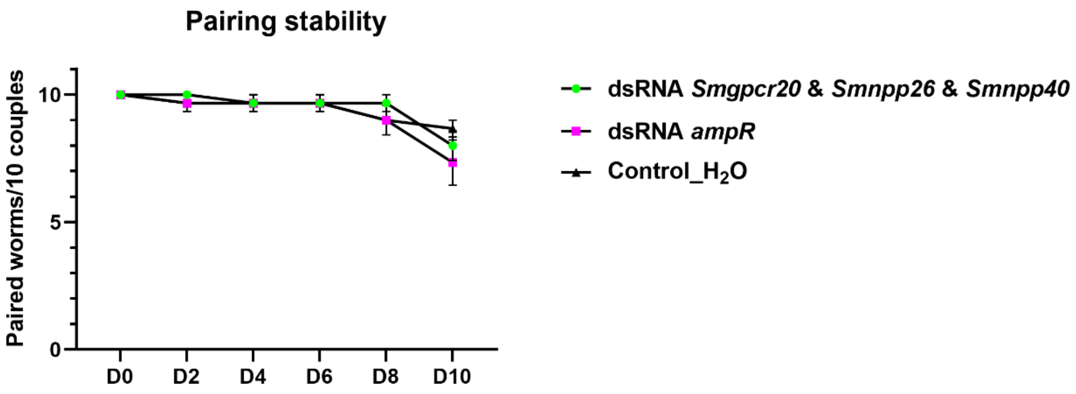

C

Only the simultaneous KD of all three genes caused significantly reduced production of normal, well-shaped eggs during the first 4 days (D2 –D4) of dsRNA of couples *in vitro* (upper figure part) and during the complete observation period (lower figure part)

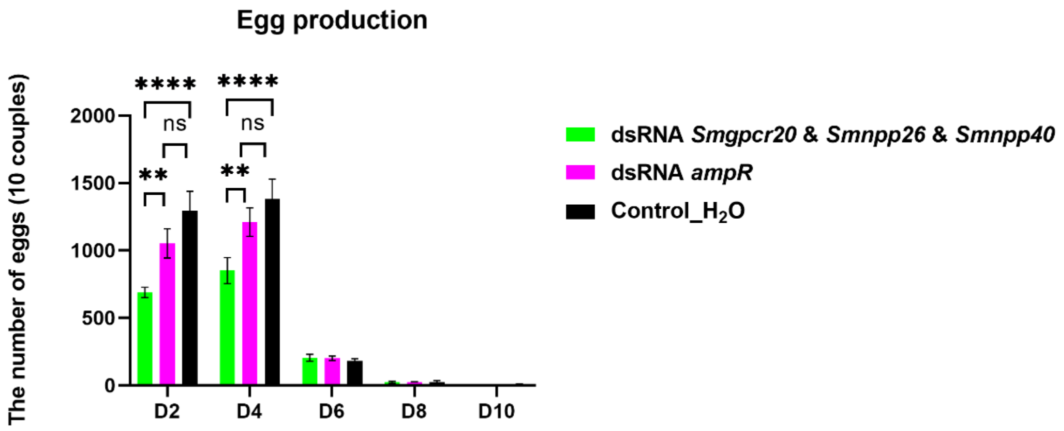

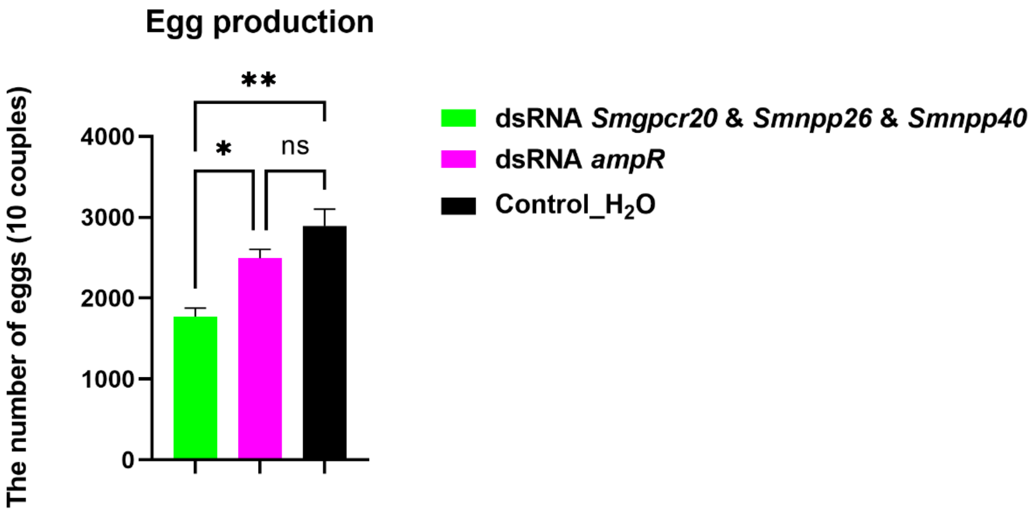

D

After about 8 d of culture, the overall number of deformed eggs increased, however, without any significant difference between the three dsRNA treatment groups. Shown are the levels of deformed eggs during (i) a 10 d observation period (upper figure part) and (ii) the total number of deformed eggs observed during the complete observation period (lower figure part)

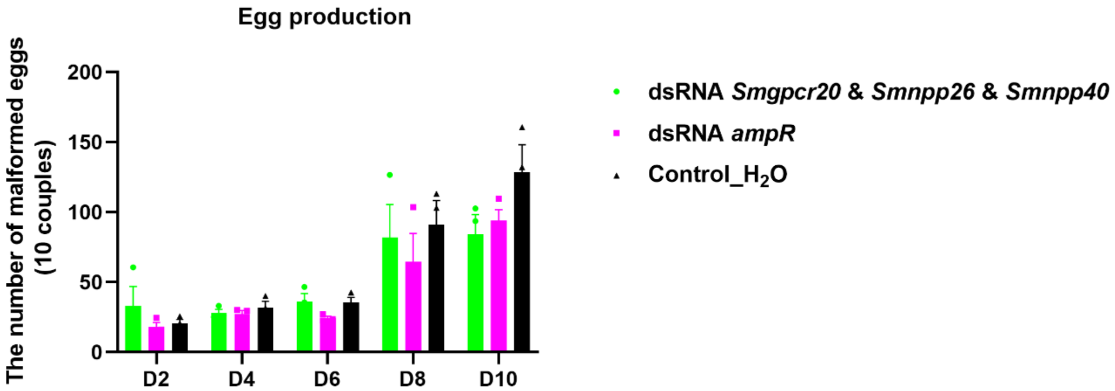

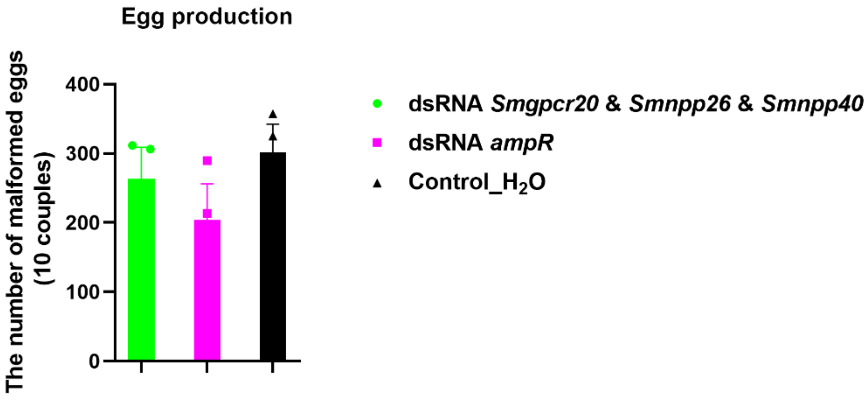

E

EdU staining of paired females showed a significant reduction of stained immature oocytes only between the respective controls and the triple KD group but not between the control groups at 15 day of the analysis

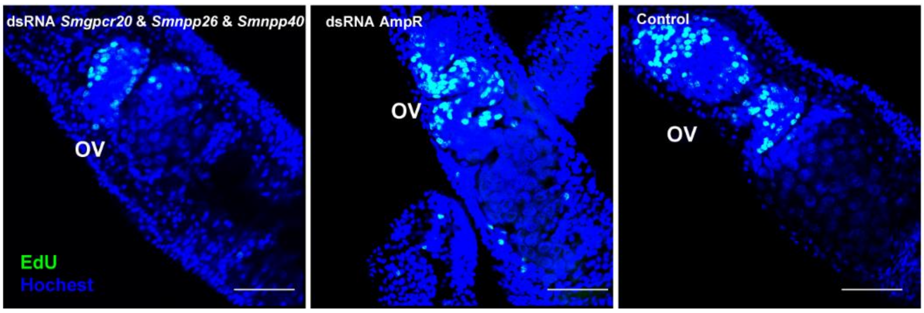

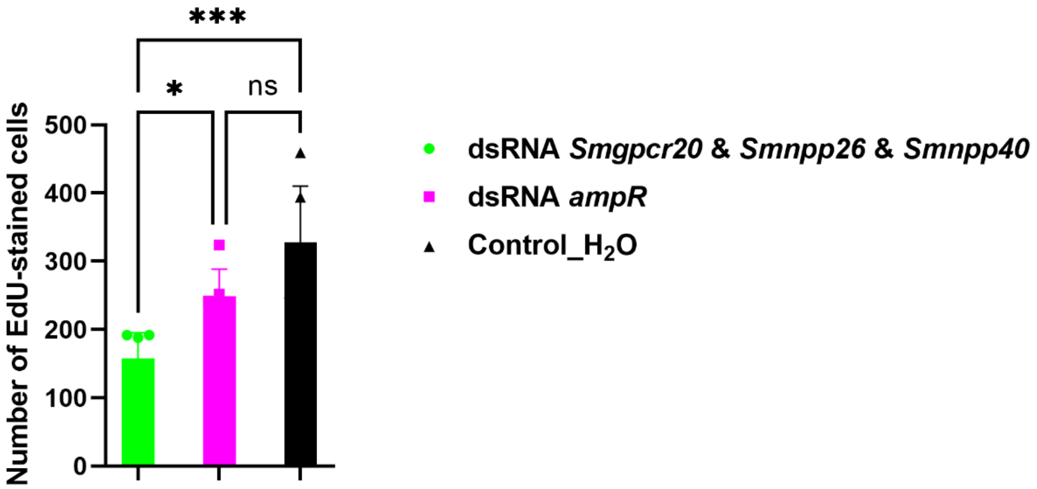

F

CLSM analysis of females showed a significantly reduced size and less oocytes only in the triple KD group but not between the control groups at 15 day of the analysis. The diagram (lower part) shows the results of the Image J-base analysis of ovary areas of 6 different paired female worms per experimental group.

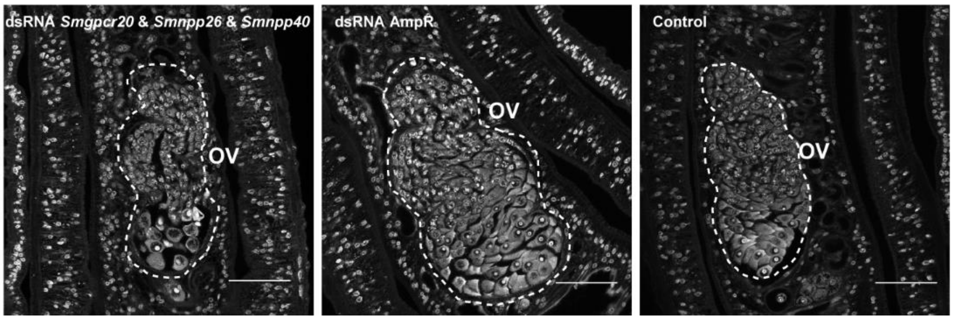

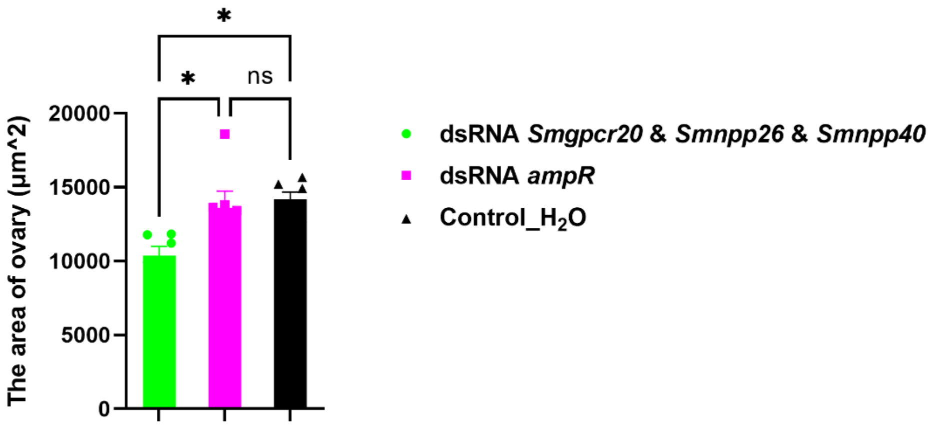

G

In first-time paired *S. mansoni* females, similar KD efficiencies of each of the three target genes were obtained (A) as with pairing-experienced females. In contrast to the triple KD group, which showed significantly smaller females, no significant length reduction of females was observed between both control groups after an observation period of 21 days (B)

A

***Smgpcr* 20**

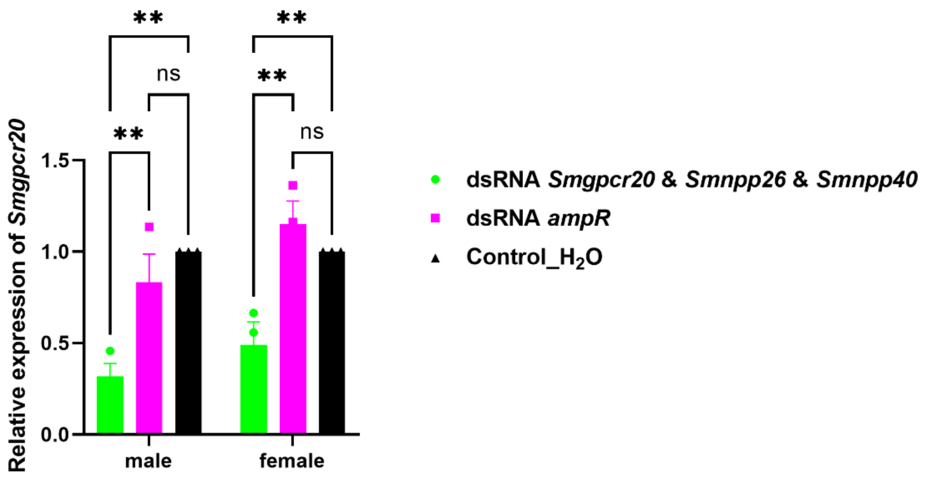

Smnpp26

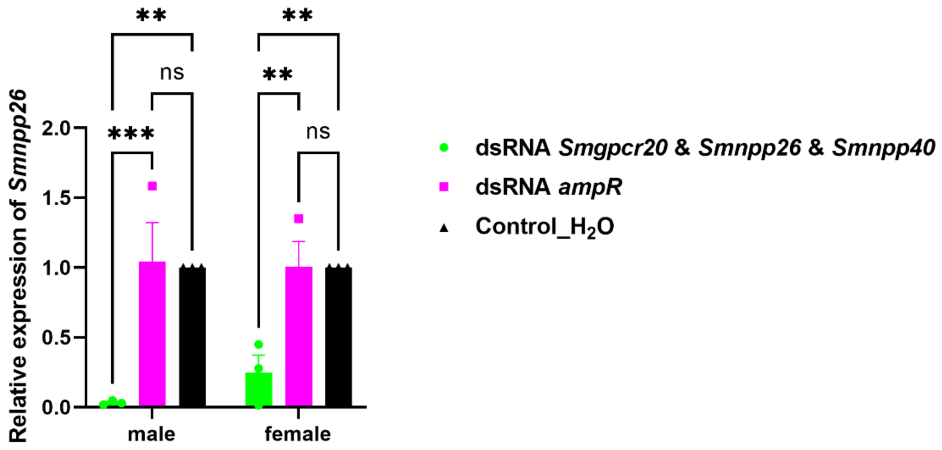

Smnpp40

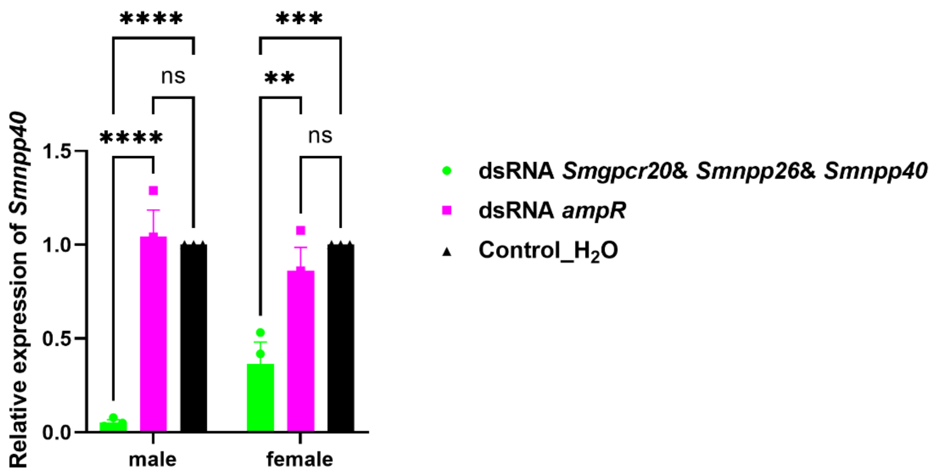

B

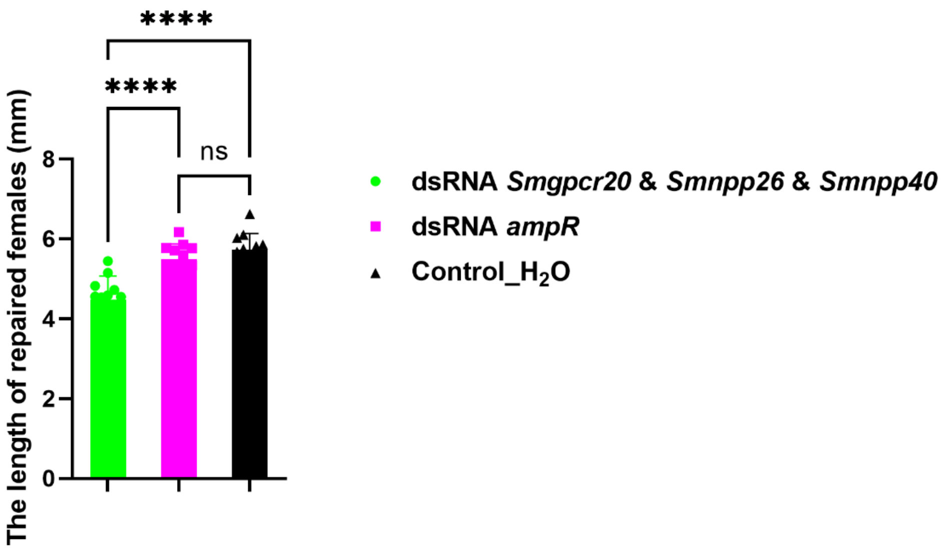

Suppl. Fig. S8: ****P < 0.0001, ***P < 0.001, **P < 0.01, *P < 0.05; ns, no significance; scale bare in E and F: 100 μm.

Supplementary Figure S9

RNAi-induced reduction of the transcript levels of *Smgpcr20, Smnpp26*, and *Smnpp40* in first-time paired females.

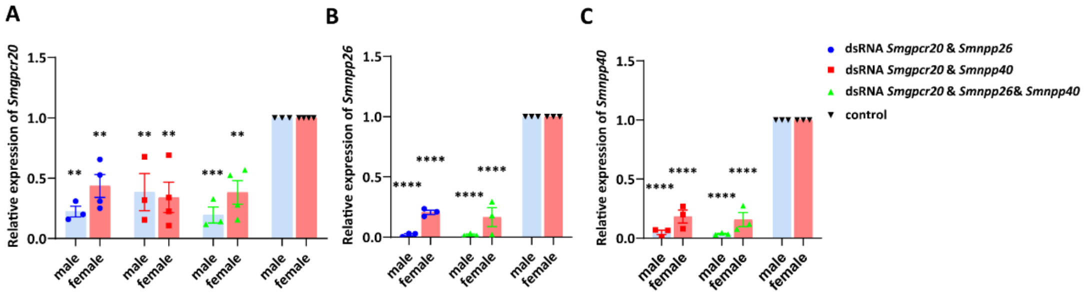

Suppl. Fig. S9: Shown is the significant reduction of the transcript levels of *Smgpcr20*, *Smnpp26*, and *Smnpp40* upon dsRNA treatment *in vitro*. The bars indicate the relative transcript levels of *Smgpcr20* (**A**), *Smnpp26* (**B**), and *Smnpp40* (**C**), respectively, in *S. mansoni* males and females after treatment with dsRNA combinations of *Smgpcr20* and *Smnpp26*, *Smgpcr20* and *Smnpp40* dsRNA, or all three specific dsRNAs for 8 d, which was followed by a 21 d culture period. Worms without dsRNA treatment served as control. After the 21 d period, the couples were separated for RNA extraction of each gender. All dsRNA-treated groups were compared with control (non-treated) groups. Fold changes of gene expression levels between dsRNA-treated worms and the control were calculated using the $2^{-\Delta\Delta Ct}$ method (96). Data are representatives of the mean ± SEM of three or four separate experiments (n = 3-4). Significant differences were determined by One-way ANOVA and indicated as: $^{****}P < 0.0001$, $^{***}P < 0.001$, $^{**}P < 0.01$.

Supplementary Figure S10

EdU staining of males following RNAi against *Smgpcr20, Smnpp26*, and *Smnpp40*

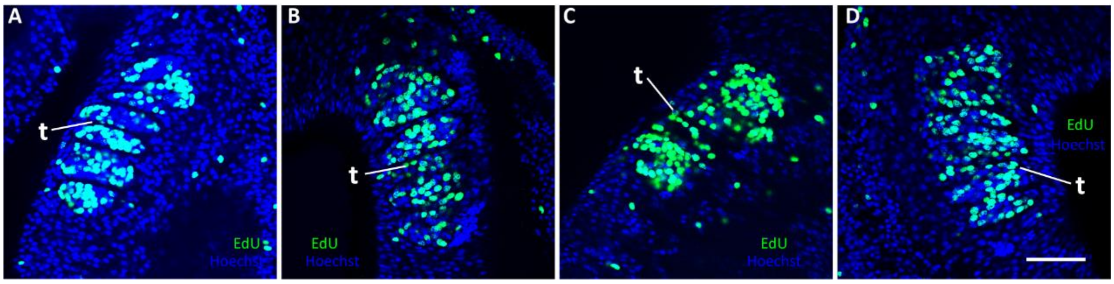

Suppl. Fig. S10: Results of the EdU-staining of male gonadal stem-cells upon dsRNA treatment. Shown are representative images of testes from the control (**D**, untreated) and dsRNA combinations of *Smgpcr20* and *Smnpp26* (**A**), *Smgpcr20* and *Smnpp40* dsRNA (**B**), or all three specific dsRNAs (**C**) males showing no differences of stem-cell proliferation following treatment. Cells were stained Hoechst 33342 (blue); EdU-stained cells were colored in green; t: teste. Scale bar: 50 µm.

Supplementary Figure S11

Double FISH analyses of *Smgpcr20*, *Smnpp26*, and *Smnpp40*

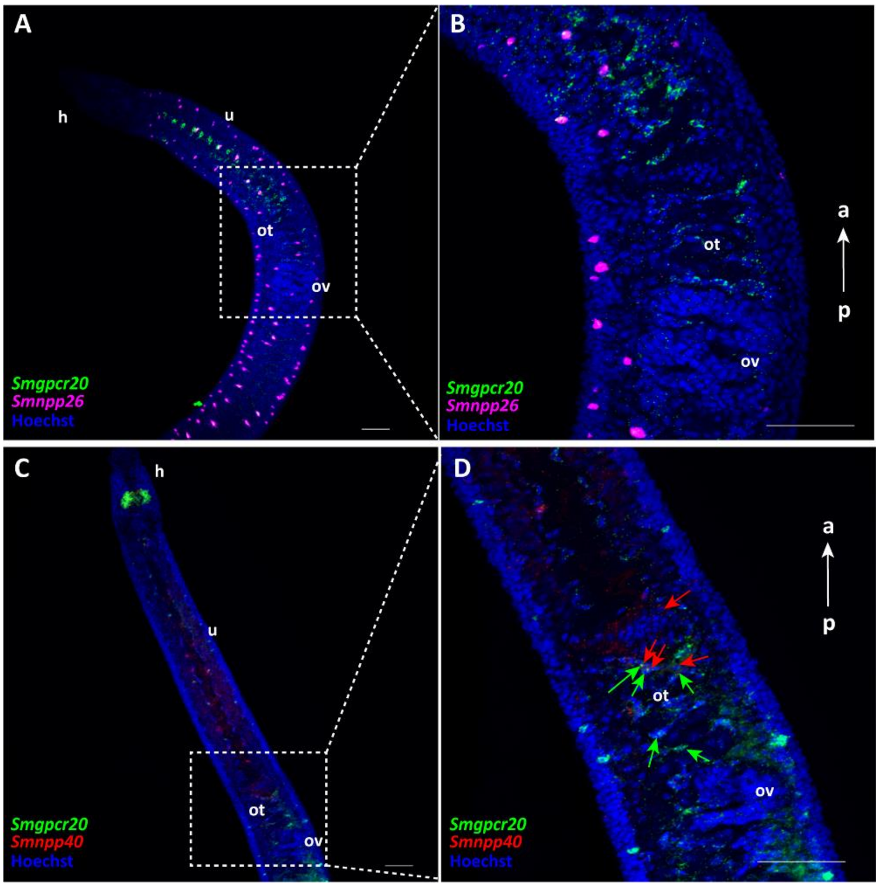

Suppl. Fig. S11: **A**, Representative images of double FISH experiments showing *Smgpcr20* and *Smnpp26* transcripts in the anterior “head” part of sF. **B**, Close-up of the ootype region of the female body showing no obvious colocalization of *Smgpcr20* and *Smnpp26*. For counter staining, cells were labelled by Hoechst 33342 (blue). FITC-labelled *Smgpcr20* transcripts are shown in green, and DIG-labelled *Smnpp26* transcripts in magenta. White arrows indicate the direction of sF from posterior (p) to anterior (a). **C**, Results of double FISH experiments showing *Smgpcr20* and *Smnpp40* transcripts in the anterior part of sF. **D**, Results of double FISH experiments showing *Smgpcr20* and *Smnpp40* transcript colocalization around the ootype of sF. Cells were labelled by Hoechst 33342 (blue), FITC-labeled *Smgpcr20* transcripts are shown in green, and DIG-labelled *Smnpp40* transcripts in red. White arrows indicate the direction of sF from posterior (p) to anterior (a). ov: ovary, u: uterus, ot: ootype, h: head. Scale bars: 50 µm.

Supplementary Table S1

Primers used to generate neuropeptide CDS

NPP	5’-3’ sequence (forward and reverse)		Length (bp)
1a	GTACCCAAACCGCTTTTGTACGTCTGGGG		29
	CCCCAGACGTACAAAAGCGGTTTGG		25
1b	GTACCCAAACCGGATTTGTTCGGATCGGT		29
	ACCGATCCGAACAAATCCGGTTTGG		25
2a	GTACCCAAACCCGAGGAATGATTGGC		26
	GCCAATCATTCCTCGGGTTTGG		22
2b	GTACCCAAACCCGAGGTTTTATGGGT		26
	ACCCATAAAACCTCGGGTTTGG		22
5a	GTACCCAAACCGCTGCTTACATGGATTTACCATGGGGT		38
	ACCCCATGGTAAATCCATGTAAGCAGCGGTTTGG		34
5b	GTACCCAAACCGCAGCTTATATTGATTTACCATGGGGT		38
	ACCCCATGGTAAATCAATATAAGCTGCGGTTTGG		34
6	GTACCCAAACCGCTGTCCGATTAATGAGACTTGGT		35
	ACCAAGTCTCATTAATCGGACAGCGGTTTGG		31
13	GTACCCAAACCCATTTTATGCCTCAACGATTTGGA		35
	TCCAAATCGTTGAGGCATAAAATGGGTTTGG		31
14	GTACCCAAACCGGATTACGTAATATGCGTATGGGT		35
	ACCCATACGCATATTACGTAATCCGGTTTGG		31
15a	GTACCCAAACCGTTCAATTTCTACGTCTTGGT		32
	ACCAAGACGTAGAAATTGAACGGTTTGG		28
15b	GTACCCAAACCTCTGCTTATCCTTATGTTGGT		32
	ACCAACATAAGGATAAGCAGAGGTTTGG		28
16	GTACCCAAACCAATTATTTATGGGATACACGTTTGGGT		38
	ACCCAAACGTGTATCCCATAAATAATTGGTTTGG		34
20a	Fw1	GTACCCAAACCGCACAAGCATTAGCTAAACTTATGTCATTATTTTATACTAGTGATGCAT	60
	Fw2	TTAATAAATATATGGAAAATCTTGATGCATATTATATGCTTAGAGGTAGACCAAGATTTGGT	62
	Re1	TTAAATGCATCACTAGTATAAAATAATGACATAAGTTTAGCTAATGCTTGTGCGGTTTGG	60
	Re2	ACCAAATCTTGGTCTACCTCTAAGCATATAATATGCATCAAGATTTTCCATATATTTA	58
20b	Fw1	GTACCCAAACCGCAGTTGAAATTGTTCCACCAGAAAGACCATTTATATTTGAAACACCTG	60
	Fw2	AAGCTCTTAGAACATATTTACATAAATTAAATGAATATTTTGCTATTATAGGTCGTCCTAGATTTGGT	68
	Re1	GCTTCAGGTGTTTCAAATATAAATGGTCTTTCTGGTGGAACAATTTCAACTGCGGTTTGG	60

	Re2 ACCAAATCTAGGACGACCTATAATAGCAAAATATTCATTTAATTTATGTAAATATGTTCTAAGA	64
23	GTACCCAAACCTATATTAGATTTGGA	26
	TCCAAATCTAATATAGGTTTGG	22
24	GTACCCAAACCGGTGGAATGTATGGTGGTCTATTAGGA	38
	TCCTAATAGACCACCATACATTCCACCGGTTTGG	34
26a	GTACCCAAACCAATTTTGATCCAATTCTGTTT	32
	AAACAGAATTGGATCAAAATTGGTTTGG	28
26b	GTACCCAAACCTCATACTTTGATCCAATTATTTAT	35
	ATAAATAATTGGATCAAAGTATGAGGTTTGG	31
26c	GTACCCAAACCTCATACTTTGATCCTATATTATTT	35
	AAATAATATAGGATCAAAGTATGAGGTTTGG	31
26d	GTACCCAAACCAATGAGGATCGTCAGTTTGAA	32
	TTCAAACTGACGATCCTCATTGGTTTGG	28
26e	GTACCCAAACCGAACATTTTGATCCGATAATTTAT	35
	ATAAATTATCGGATCAAAATGTTCGGTTTGG	31
27	GTACCCAAACCGTTCCACCTTATATAACCGGTGGAATTCGGTAT	44
	ATACCGAATTCCACCGGTTATATAAGGTGGAACGGTTTGG	40
28	GTACCCAAACCGCTTATCATTTCTTTCGATTG	32
	CAATCGAAAGAAATGATAAGCGGTTTGG	28
29	GTACCCAAACCATGGTGTATTGG	29
	CCAATACACCATGGTTTGG	19
32.1A	GTACCCAAACCGGTCCAGAAACACTTTGGGAACTGGAC	38
	GTCCAGTTCCCAAAGTGTTTCTGGACCGGTTTGG	34
32.1B	GTACCCAAACCGGTCCAGAACCATTATGGGTAGTAGAAACT	41
	AGTTTCTACTACCCATAATGGTTCTGGACCGGTTTGG	37
32.2	GTACCCAAACCGGTCCAGAATTAATTATTCCATTTATAAGTGGCGGTGTTCCAGCA	56
	TGCTGGAACACCGCCACTTATAAATGGAATAATTAATTCTGGACCGGTTTGG	52
35.1	GTACCCAAACCTATGGACATTATTCACAACGTTTAGGA	38
	TCCTAAACGTTGTGAATAATGTCCATAGGTTTGG	34
35.2	GTACCCAAACCTATTATATATCACAAAGACTTGGT	35
	ACCAAGTCTTTGTGATATATAATAGGTTTGG	31
36.1	GTACCCAAACCTGGTTTCCTATAAAAGAATATCGTGGTGGATTAATGGAAGTT	53
	AACTTCCATTAATCCACCACGATATTCTTTTATAGGAAACCAGGTTTGG	49
36.2a	GTACCCAAACCTGGTATCCTGTGAAAGAATTTCATTATGATGAACCGTTAGAGATT	56
	AATCTCTAACGGTTCATCATAATGAAATTCTTTCACAGGATACCAGGTTTGG	52
36.2b	GTACCCAAACCTGGTTTCCAGTGAAAGAATTCCATTATGATGGACCACTTGAAGTG	56

	CACTTCAAGTGGTCCATCATAATGGAATTCTTTCACTGGAAACCAGGTTTGG	52
36.2c	GTACCCAAACCTGGTCTCCTGTCAAAGAATTTCATTATGATGAACCAATAGAAGTG	56
	CACTTCTATTGGTTCATCATAATGAAATTCTTTGACAGGAGACCAGGTTTGG	52
37	GTACCCAAACCTGGACTGATTTT	23
	AAAATCAGTCCAGGTTTGG	19
38a	GTACCCAAACCGTTTTAGCTGATTAT	26
	ATAATCAGCTAAAACGGTTTGG	22
38b	GTACCCAAACCCAAGCTATATTAGCTGATTAC	32
	GTAATCAGCTAATATAGCTTGGGTTTGG	28
39	GTACCCAAACCTTCACTCGTCCATATGGT	29
	ACCATATGGACGAGTGAAGGTTTGG	25
40a	GTACCCAAACCTTTCTGTTAGCTTTACCGTCACCC	35
	GGGTGACGGTAAAGCTAACAGAAAGGTTTGG	31
40b	GTACCCAAACCTTTCTACTTGGTCTACCGCCTAAAGTTGAACAT	44
	ATGTTCAACTTTAGGCGGTAGACCAAGTAGAAAGGTTTGG	40
40c	GTACCCAAACCTTTCTACTTGGTTTACCACCATCACTTAGACAACAT	47
	ATGTTGTCTAAGTGATGGTGGTAAACCAAGTAGAAAGGTTTGG	43
40d	GTACCCAAACCTTCATTTTAGGGCTACCAGCACCAACTAGATTTCATTCG	50
	CGAATGAAATCTAGTTGGTGCTGGTAGCCCTAAAATGAAGGTTTGG	46
41	GTACCCAAACCTTCTTTTGTAATCCAATGGGATGCGTT	38
	AACGCATCCCATTGGATTACAAAAGAAGGTTTGG	34
42	GTACCCAAACCCCTTGGACATTACGTGACCCACTGAATTGTTGCTTGGATAATGCTAAATGTTGT	65
	ACAACATTTAGCATTATCCAAGCAACAATTCAGTGGGTCACGTAATGTCCAAGGGGTTTGG	61
43a	GTACCCAAACCGCAAGTTTAGCATATTTT	29
	AAAATATGCTAAACTTGCGGTTTGG	25
43b	GTACCCAAACCGCAAGTTTATCCTATTTT	29
	AAAATAGGATAAACTTGCGGTTTGG	25
47	GTACCCAAACCGGCAAATTTTTCATGTTAGGA	32
	TCCTAACATGAAAAATTTGCCGGTTTGG	28
48	CGTTGTGGGTACCCAAACCTATTATACAAATTTGAAAACAATTG	44
	GGTCGTACCAGATCCCCCACCATATCTCATCACATTAGG	39

Supplementary Table S2

Primers used for GPCR gene amplification

**GPCR**	**5'-3' sequence (forward and reverse)**	**Length (bp)**
Smp_084270 (*Sm*GPCR20)	CCGCCCAATACGAGCCCATGATAAGTATGAACTCAAGTGAATT	43
	GTTGATCCACCTTCTAGGATCCCGTAATTGTGGCCTGATACAACG	45

Supplementary Table S3

Primers used for pJC 53.2 plasmids construction and qPCR

Primer name	Application	Sequence (5'-3')
Smp_084270 (*Smgpcr20*) _ pJC 53.2_s	Cloning	ATACGGCTTGCAATGTTGGG
Smp_084270 (*Smgpcr20*) _ pJC 53.2_as	Cloning	TGTGGCCTGATACAACGCTT
Smp_084270 (*Smgpcr20*) _ qPCR_For	qRT-PCR	CCGTATACGACAAATGGAACC
Smp_084270 (*Smgpcr20*) _ qPCR_Rev	qRT-PCR	TCGGATGAAGCACATACAC
Smp_084270 (*Smgpcr20*) _T7_For	dsRNA	cctaatacgactcactatagggagCGATTACTGCATGCCGCTTT
Smp_084270 (*Smgpcr20*) _T7_Rev	dsRNA	cctaatacgactcactatagggagTGTTGGGTTCAGAGTGCCAA
Smp_084270 (*Smgpcr20*) _ qPCR_For	qRT-PCR(RNAi)	CCGTATACGACAAATGGAACC
Smp_084270 (*Smgpcr20*) _qPCR_Rev	qRT-PCR(RNAi)	TCGGATGAAGCACATACACG
Smp_071050 (*Smnpp26*) _ pJC 53.2_s	Cloning	TCGTCAATGCTATACCTGTGC
Smp_071050 (*Smnpp26*) _ pJC 53.2_as	Cloning	TTCATTGATTACATTGTGCGTCT
Smp_071050 (*Smnpp26*) _ qPCR_For	qRT-PCR	TGGGTTTTCATGGGTTGCAAG
Smp_071050 (*Smnpp26*) _ qPCR_Rev	qRT-PCR	TACACCTCCACCAATTCCGC
Smp_004710 (*Smnpp40*) _ pJC 53.2_s	Cloning	GTTTATTACTTACCCCTCCTCCA
Smp_004710 (*Smnpp40*) _ pJC 53.2_as	Cloning	GTTCAACTTTAGGCGGTAGACC
Smp_004710 (*Smnpp40*) _ qPCR_For	qRT-PCR	GGTCTACCGCCTAAAGTTGAAC
Smp_004710 (*Smnpp40*) _ qPCR_Rev	qRT-PCR	TGAAATCTAGTTGGTGCTGGT
Smp_335630 (*Smtsp-2*) _ pJC 53.2 _s	Cloning	CTCTTGGTTGTGGGTATAAG
Smp_335630 (*Smtsp-2*) _ pJC 53.2_as	Cloning	CATGTTCGTCATTACGGTAC
Smp_065110 (*Smletm1*) _qPCR_For	qRT-PCR	CGTGGAATGCGTTCAGTTGG
Smp_065110 (*Smletm1*) _qPCR_ Rev	qRT-PCR	GAAGCTGATGGAGGTAATTGAG

Smp_055740 (*Smnanos-1*) _qPCR_For	qRT-PCR	ACTTGTCCATTATGCGGTGCT
Smp_055740 (*Smnanos-1*) _qPCR_Rev	qRT-PCR	GGTTCCAACAAACCAGCTTCA
Smp_051920 (*Smnanos-2*) _qPCR_For	qRT-PCR	GCCGTGTTATGACCTCTGG
Smp_051920 (*Smnanos-2*) _qPCR_Rev	qRT-PCR	GACGATCTGGAGACTCTGG
Smp_000270 (*Smfs800*) _qPCR_For	qRT-PCR	CAGCCGAAAAAGTCAAACA
Smp_000270 (*Smfs800*) _qPCR_Rev	qRT-PCR	CCCTTTTGCATCGTAAGCT
Smp_333540 (*Smmeiob*) _ qPCR_For	qRT-PCR	TGCTGGATATGCCTGTACATTCGCC
Smp_333540 (*Smmeiob*) _ qPCR_Rev	qRT-PCR	ACCTGGAGTAGCGCACATTGCAAAC
Smp_050270 (*Smtyr1*) _qPCR_For	qRT-PCR	AGTATGCGGTGGACCAAAAC
Smp_050270 (*Smtyr1*) _qPCR_Rev	qRT-PCR	ATCGTCCTTTCCATCCAAAC
Smp_013540 (*Smtyr2*) _ qPCR_For	qRT-PCR	ACAGCATTCCCAACAACTCA
Smp_013540 (*Smtyr2*) _ qPCR_Rev	qRT-PCR	CACCGGGAAAAGAACAAAAT
Smp_165360 (*Smmyst4*) _qPCR_For	qRT-PCR	GAAATTCGTTTCCCAAGCAG
Smp_165360 (*Smmyst4*) _qPCR_Rev	qRT-PCR	GCCCCTTCGTAGCCATTTA
Smp_165360 (*Smmyst4*) _ pJC 53.2_s	Cloning	CCACATATCAAGCAAAGTCAACTGCTGAA
Smp_165360 (*Smmyst4*) _ pJC 53.2_as	Cloning	CAGTTGGTTGACTTGATGAAATAAGCTCATC
Smp_131110 (*Smp14*) _qPCR_For	qRT-PCR	CCTATGGCGGTGATTATGG
Smp_131110 (*Smp14*) _qPCR_Rev	qRT-PCR	GGCTGGGTTTGTAAGTGC
Smp_095980 (*Smsod*)_ qPCR_For	qRT-PCR	TTTGATCCGGCTATTGCTTC
Smp_095980 (*Smsod*)_ qPCR_Rev	qRT-PCR	TCATGGTGCACGAAATCCTA
Ampicillin_ pJC 53.2_s	cloning	GAGTATTCAACATTTCCGTGTCGC
Ampicillin_ pJC 53.2_as	cloning	CGGTTCCCAACGATCAAGGC
pJC 53.2_sequence_s	Cloning	TTCTGCGGACTGGCTTTCTAC
pJC 53.2_T7_extended primer	Riboprobe synthesis	CCTAATACGACTCACTATAGGGAG

*Sequence of the T7 promoter: lower case letters.
